# Bergenin mitigates neuroinflammatory damage induced by high glucose: insights from Zebrafish, murine microbial cell line, and rat models

**DOI:** 10.3389/fphar.2024.1339178

**Published:** 2024-08-01

**Authors:** Wenjing Yu, Rongsiqing Luo, Chunxiang He, Ze Li, Miao Yang, Jinyong Zhou, Jiawei He, Qi Chen, Zhenyan Song, Shaowu Cheng

**Affiliations:** ^1^ School of Integrated Chinese and Western Medicine, Hunan University of Chinese Medicine, Changsha, Hunan, China; ^2^ Key Laboratory of Hunan Province for Integrated Traditional Chinese and Western Medicine on Prevention and Treatment of Cardio-Cerebral Diseases, School of Integrated Chinese and Western Medicine, Hunan University of Chinese Medicine, Changsha, Hunan, China; ^3^ The First Hospital of Hunan University of Chinese Medicine, Changsha, Hunan, China; ^4^ Department of Science and Technology, Hunan University of Chinese Medicine, Changsha, Hunan, China

**Keywords:** bergenin, diabetes-associated cognitive impairment (DACI), glycolysis, neuroinflammation, Zebrafish

## Abstract

**Background:**

The escalating global burden of diabetes and its associated cognitive impairment underscores the urgency for effective interventions. Bergenin shows promise in regulating glucose metabolism, mitigating inflammation, and improving cognitive function. Zebrafish models offer a unique platform for assessing drug efficacy and exploring pharmacological mechanisms, complemented by subsequent investigations in cell and rat models.

**Methods:**

The experimental subjects included zebrafish larvae (CZ98:*Tg (mpeg1:EGFP)* ^*ihb20Tg/+*^), adult zebrafish (immersed in 2% glucose), BV2 cell line (50 mM glucose + 10 μm Aβ_1-42_), and a streptozotocin (STZ) bilateral intracerebroventricular injection rat model. Bergenin’s effects on the toxicity, behavior, and cognitive function of zebrafish larvae and adults were evaluated. The Morris water maze assessed cognitive function in rats. Neuronal histopathological changes were evaluated using HE and Nissl staining. qPCR and Western blot detected the expression of glycolysis enzymes, inflammatory factors, and Bergenin’s regulation of PPAR/NF-κB pathway in these three models.

**Results:**

1) In zebrafish larvae, Bergenin interventions significantly reduced glucose levels and increased survival rates while decreasing teratogenicity rates. Microglial cell fluorescence in the brain notably decreased, and altered swimming behavior tended to normalize. 2) In adult zebrafish, Bergenin administration reduced BMI and blood glucose levels, altered swimming behavior to slower speeds and more regular trajectories, enhanced recognition ability, decreased brain glucose and lactate levels, weakened glycolytic enzyme activities, improved pathological changes in the telencephalon and gills, reduced expression of pro-inflammatory cytokines, decreased *ins* expression and increased expression of *irs1*, *irs2a*, and *irs2b*, suggesting a reduction in insulin resistance. It also altered the expression of *pparg* and *rela*. 3) In BV2 cell line, Bergenin significantly reduced the protein expression of glycolytic enzymes (GLUT1, HK2, PKFKB3, and PKM2), lowered IL-1β, IL-6, and TNF-α mRNA expression, elevated PPAR-γ protein expression, and decreased P-NF-κB-p65 protein expression. 4) In the rat model, Bergenin improves learning and memory abilities in STZ-induced rats, mitigates neuronal damage in the hippocampal region, and reduces the expression of inflammatory factors IL-1β, IL-6, and TNF-α. Bergenin decreases brain glucose and lactate levels, as well as glycolytic enzyme activity. Furthermore, Bergenin increases PPARγ expression and decreases p-NF-κB p65/NF-κB p65 expression in the hippocampus.

**Conclusion:**

Bergenin intervenes through the PPAR-γ/NF-κB pathway, redirecting glucose metabolism, alleviating inflammation, and preventing high glucose-induced neuronal damage.

## 1 Introduction

According to a report from the International Diabetes Federation (IDF), in 2019, the global count of adult diabetes patients reached 463 million. Projections suggest this number will rise to 578 million by 2030 and surge to 700 million by 2045 (Sun et al., 2022). Diabetes-Associated Cognitive Impairment (DACI) emerges as a chronic complication of type 2 diabetes (T2DM), manifesting in diminished memory, comprehension, and spatial orientation abilities, significantly impacting patients’ self-care and quality of life. DACI has now surpassed cardiovascular complications, becoming the second leading cause of death among diabetic patients (Bellia et al., 2022; Li et al., 2023). Meanwhile, epidemiological studies indicate that T2DM can elevate the risk of Alzheimer’s disease (AD) by 1.5–2.5 times (Xue et al., 2019). The primary pathogenic mechanism of DACI involves disrupted cerebral glucose metabolism, leading to overactivation of microglial cells, resulting in neuroinflammation and subsequent cognitive impairment (Dove et al., 2021; Leng and Edison, 2021). Despite this understanding, the precise mechanism linking cognitive decline and T2DM remains incompletely elucidated, with no available drugs to effectively halt disease progression. Given the crucial role of neuroinflammation induced by activated microglial cells in DACI pathogenesis, regulating glucose metabolism to prevent excessive microglial cell activation represents a novel approach for early DACI intervention.

Compared to synthetic drugs, natural products have increasingly attracted attention as therapeutic agents due to their lower cytotoxicity and reduced side effects (Zhu et al., 2022), some natural products have demonstrated significant efficacy in improving diabetes and its complications, as well as in reducing inflammatory responses (Sharma et al., 2020). *Bergenia purpurascens* (Hook. f. et Thoms.) Engl., a plant belonging to Saxifragaceae, Bergenia Moench, is used in traditional Chinese medicine. Its primary active component, bergenin, a C-glycoside of 4-O-methylgallic acid, exhibits diverse pharmacological effects, including the regulation of glucose and lipid metabolism, anti-inflammatory properties, and mitigation of oxidative stress (Barai et al., 2019; Villarreal et al., 2020; Zhang et al., 2023). Numerous studies have demonstrated bergenin’s role in regulating glucose metabolism and improving diabetes and its complications (Barai et al., 2019; Qiao et al., 2019; Villarreal et al., 2020; Yin et al., 2023; Zhang et al., 2023). Recent research has identified bergenin as a promising therapeutic agent for inhibiting glycolysis by downregulating Hexokinase 2 (HK2), the first glycolytic rate-limiting enzyme (Li et al., 2023). Furthermore, bergenin modulates the production of pro- and anti-inflammatory cytokines, reducing the expression of IL-1β, IL-6, and TNF-α proinflammatory cytokines, thereby ameliorating inflammatory responses (Yin et al., 2023). Additionally, bergenin exerts neuroprotective effects, improving cognitive dysfunction (Ji et al., 2019; Singla et al., 2022).

Zebrafish offer distinct advantages in studying mechanisms of glucose metabolism, sharing biological mechanisms with humans in regulating glucose homeostasis (Zang et al., 2017). Key metabolic organs and genes are conserved in zebrafish (Cox et al., 2018), which exhibit hyperglycemic symptoms and impaired glucose metabolism when fed a glucose-rich diet (Kleinert et al., 2018). The zebrafish model of type II diabetes mellitus responds positively to antidiabetic drugs like metformin and glimepiride, making them valuable models for investigating human diabetes and metabolic disorders (Mohammadi et al., 2020). Furthermore, the skin or gills of zebrafish provide a gateway for non-invasive administration of biologically active compounds, allowing precise delivery into the water surrounding hundreds of zebrafish embryos, larvae, or adults (Angom and Nakka, 2024). This feature makes zebrafish an independent system for screening antidiabetic drugs, validating their effects on type 2 diabetes mellitus (T2DM) complications, and studying pharmacokinetics (Wang et al., 2023).

The peroxisome proliferator-activated receptors (PPARs) are members of the nuclear receptor superfamily. PPARγ, a ligand-dependent nuclear transcription factor, translocates into the nucleus and binds to PPAR response elements (PPREs) upon ligand binding, thereby regulating the transcription and translation of downstream target genes involved in lipid and glucose metabolism as well as inflammation (Chen et al., 2023). This renders PPARγ an attractive pharmacological target for treating metabolic diseases such as insulin resistance, type 2 diabetes (Shehnaz et al., 2023), chronic inflammation, and degenerative disorders (Geng et al., 2018). NF-κB serves as a pivotal transcription factor in inflammation regulation. The nuclear translocation of NF-κB heterodimers plays a crucial role in microglial cell activation, induced by pro-inflammatory stimuli such as IL-1β, IL-6, and TNF-α (Poma, 2020; Peng et al., 2022). It is noteworthy that multiple experiments have demonstrated Bergenin’s role as a natural PPARγ agonist. The activation of PPARγ inhibits IκBα degradation, p65 nuclear translocation, DNA-binding activity, and phosphorylation, or indirectly inhibits NF-κB activation by competitively binding to p65, thereby reducing the production of pro-inflammatory cytokines and chemokines (Wang et al., 2017; Yang et al., 2022).

Given the pivotal roles of glucose metabolism disorder and neuroinflammation in diabetic neuropathy, this study aims to confirm bergenin’s potential protective effects against high glucose-induced glycolysis enhancement and neuroinflammatory responses, while preliminarily exploring its pharmacological mechanisms. This study employs the zebrafish model for rapid assessment of drug safety and validation of pathological mechanism hypotheses. Subsequently, a comprehensive investigation into the molecular mechanisms was conducted using rat and cell models (Supplementary Figures S1, 2).

## 2 Materials and methods

### 2.1 Experimental animals and cells

Wild-type zebrafish (AB line, *Danio rerio*) and transgenic zebrafish (CZ98:*Tg (mpeg1:EGFP)* ^*ihb20Tg/+*^) were obtained from the China Zebrafish Resource Center and bred at the Zebrafish Breeding Platform of the Hunan Key Laboratory for Integrative Prevention and Treatment of Cardio-Cerebral Diseases, Hunan University of Chinese Medicine. Zebrafish were maintained in tanks with a water temperature of 28°C ± 0.5°C, pH 7.0–7.3, dissolved oxygen 7.3 ± 0.2 mg/L, conductivity 460 ± 50 μS/cm, water hardness 128 ± 25 mg/L, and salinity of 0.3 ng/L (Shang et al., 2024), under a light-dark cycle of 14 h:10 h with eight fish reared in each 1-L tank. They were fed twice daily at 08:30 and 17:30 with newly hatched *Artemia salina*. Embryos were obtained through natural breeding. Fertilized eggs were collected and incubated at 28°C in an incubator. All research protocols were approved by the Experimental Animal Ethics Committee of Hunan University of Chinese Medicine (Changsha, China), Approval number: LLBH-202205030001.

The BV2 cell line was purchased from Wuhan PuNuoSai Biotechnology Co., Ltd. and cultured in RPMI 1640 medium containing 10% fetal bovine serum and 1% penicillin-streptomycin at 37°C under 5% CO_2_ (He et al., 2024).

SPF-grade male SD rats (n = 50), weighing (150 ± 20) g, were purchased from Hunan Slaike Jingda Experimental Animals Co., Ltd. (License No.: SCXK(Hunan)2021–0004). The rats were housed in the SPF-grade animal barrier system at the Animal Experimental Center of Hunan University of Chinese Medicine, with three rats per cage. They were fed standard animal feed and water, maintained at a constant temperature of (25 ± 2)°C, and subjected to a 12-h light-dark cycle. The experimental procedures complied with ethical standards for animal research (LLBH202205100001).

### 2.2 Drugs and reagents

Bergenin (MedChemExpress, HY-N0017); Metformin Hydrochloride, Hexokinase (HK), Phosphofructokinase (PFK), Pyruvate Kinase (PK) Activity Assay Kit (Beijing Solarbio Science & Technology Co., Ltd., SM9400, BC0745, BC0535, BC0545); Glucose, Lactic Acid assay kit (Nanjing Jiancheng Biotech Co., Ltd., A154-1-1, A019-2-1); β-amyloid peptides (Shanghai Aladdin Bio-Chem Technology Co., Ltd., B111464); 2-DG, DADA, DMSO (Sigma-Aldrich, D8375-1G, D135665, D2650-100 ML); Fetal bovine serum, RPMI 1640 medium (Gibc, 10099-141, 11875093); D-glucose solution, Penicillin-Streptomycin Solution (Wuhan Pricella Biotechnology Co.,Ltd., PB180418, PB180120); Serum-free freezing medium, β-actin, IL-1β, IL-6, TNF-α primers (Shanghai BioWork Biotech Co., Ltd., 05-065-1B); TRIzol Reagent (Thermo Fisher Scientific, 15596018); Isopropyl alcohol (Macklin, I811925); Reverse transcription kit (Suzhou Jinkang Protein Technology Co., Ltd., 0521751); SYBR Green Premix (Shanghai MoNa Biotechnology Co., Ltd., MQ00401); DEPC-treated Water (Beyotime, R0022); PPAR gamma Antibody, NF-κB p65 Polyclonal Antibody (Bioss, BS-0530R, RRID:AB_10860216, BS-0465R, RRID:AB_10855447); Anti-RELA (Phospho-Ser276) rabbit polyclonal antibody (Sangon Biotech, D155005); β-actin (Affinity Biosciences, AF7018, RRID:AB_2839420); Goat anti-rabbit secondary antibody (Sigma-Aldrich, AP132P); RIPA Lysis Buffer (Cwbio, CW233S); BCA Protein Quantification Kit (Multi Sciences, PQ0012); Streptozotocin (STZ, Sigma-Aldrich, S0130).

### 2.3 Experimental instruments

ZebHigh-throughput Observation Chamber for Zebrafish Embryo Larvae, Automated Zebrafish Analysis System for Adult Fish Observation Tower (Viewpoint, France); Conventional Brightfield Microscope (Leica, S9i); BioTek Cytation™ 5 Cell Imaging Multi-Mode Reader (Agilent Technologies Co., Ltd., China); DW-2000D Brain Locating Instrument (Chengdu Techman Software Co., Ltd.); Smart 3.0-Video Tracking System (Panlab, RRID:SCR_002852); Tri-Gas Incubator (Thermo Fisher Scientific, United States); Inverted Fluorescence Microscope (Zeiss, Germany); SorvallTM LegendTM Micro 17R Microcentrifuge (Thermo Fisher, United States); Cytation3 Multimode Reader (Bio-Tek, United States); T100TM Thermal Cycle, CFX96 Touch Real-Time PCR Detection System (Bio-Rad, United States); Mini-PROTEAN Tetra Electrophoresis System, Mini Trans-Blot Transfer System (Bio-Rad, United States); ChemiDoc XRS + Chemiluminescence Gel Imaging System (Bio-Rad, United States of America); HM 325 Paraffin microtome (Thermo Fisher, United States); Tissue-FAXS Plus Panoramic Tissue Scanning Imaging System (Tissue Gnostics GmbH, Austria).

### 2.4 Zebrafish experiment

#### 2.4.1 Embryo-Larvae rearing and experimental grouping

The macrophage-specific GFP transgenic zebrafish (CZ98:*Tg(mpeg1:EGFP)* ^*ihb20Tg/+*^) were selected for the experiment. Healthy zebrafish embryos at 9 h post-fertilization (hpf) were randomly allocated to 6-well plates, with 30 embryos per well. Based on preliminary findings on glucose, bergenin, and metformin concentrations, the embryos were divided into four groups: Control (0.1% DMSO), Model (1% glucose + 0.1% DMSO), Bergenin (1% glucose + 2.5 mg/L bergenin), and Metformin (1% glucose + 3 mg/L metformin). Bergenin and metformin were dissolved in 0.1% DMSO for administration. Each group had three replicate wells. The plates were incubated in a constant temperature light incubator at 28°C ± 0.5°C, under a light-dark cycle of 14 h:10 h, with the solution changed every 24 h.

#### 2.4.2 Hatchability, survival rate, and teratogenicity analysis

The hatching rate was calculated on day 5 post-fertilization (dpf). Zebrafish embryos were examined for abnormalities such as organ edema and spinal curvature. Survival rate was determined on day 8 post-fertilization after larval rearing. (Li et al., 2022).Tissues were phosphate-buffered saline (PBS)-washed, homogenized at 0.1 g per sample with 1 mL extraction buffer on ice, then centrifuged at 8000 *g* for 10 min at 4°C. The resulting supernatant was collected and stored on ice for subsequent analysis. Glucose content in the supernatant was assessed following assay kit instructions. Prompt removal of detached embryo membranes and dead larvae was ensured.

#### 2.4.3 Zebrafish Larval brain fluorescence signal recording

Microglial cell activation in the brain was assessed by directly detecting fluorescence in the macrophage-specific GFP transgenic zebrafish (96hpf) using a fluorescence microscope (Bruzzone et al., 2021). The intensity and aggregation of green fluorescent signals within the brain can directly reflect the degree of microglial cell activation, thereby indirectly indicating the extent of inflammation response in the brain.

#### 2.4.4 Zebrafish Larval behavioral experiment

The locomotion patterns of zebrafish larvae were observed on the 5 th day post modeling administration. Zebrafish were grouped and placed in a 12-well plate containing 1 mL of embryo medium before being transferred to the Zebrafish High-throughput Observation Chamber. Videos and images capturing locomotion trajectories were recorded to quantify the distance (mm) and speed of movement for each fish. Movement was categorized into high-speed (>10 mm/s), medium-speed (2–10 mm/s), and low-speed (<2 mm/s) to determine their states. Zebrafish locomotion distance and trajectories were recorded and analyzed for 60 s in this experiment (Benvenutti et al., 2021).

#### 2.4.5 The grouping and treatment of adult zebrafish experiments

A total of 240 adult zebrafish (6–8 months old) were randomly assigned to control, model, and three bergenin dose groups (1.25 mg/L, 2.5 mg/L, and 5 mg/L), as well as a metformin group (3 mg/L), with each group containing 40 fish. The control group remained in standard water conditions, while the model, bergenin, and metformin groups were immersed in a 2% glucose solution (10 fish per 1500 mL) for 28 days. (Chen and Liu, 2022). Fish were fed three times the standard amount of Artemia daily to induce hyperglycemia. Starting from day 21, the low, medium, and high-dose bergenin groups, along with the metformin group, received continuous administration for 7 days. Measurements of body length, weight, body mass index (BMI), and blood glucose levels were obtained via tail clipping. Half of the solution volume in each tank was replaced daily.

#### 2.4.6 Zebrafish vitality and T-maze behavioral testing experiment

Following drug administration, behavioral and T-maze experiments were conducted on zebrafish to observe changes and assess the efficacy of the model and medication. Zebrafish behavioral tests were conducted in a square tank, with each fish observed for 180 s per trial over three consecutive days. This method serves to assess zebrafish vitality and anxiety levels (Wang et al., 2023). Furthermore, the T-maze was used to evaluate zebrafish learning and memory. The T-maze consisted of horizontally equal-length left and right arms and a vertical channel. Specifically, the left arm was designated as the Enriched Chamber (EC) area, where specific enrichment was provided. The starting point was located at one end of the vertical channel, which corresponds to the end of the non-equal length left and right arms. The testing period spanned 5 days.

Formal testing began promptly at 09:00 each morning. Prior to the start, a single zebrafish designated for testing was placed in the starting zone. It was allowed 1 min to acclimate to the T-maze environment, including water quality and temperature, with the starting zone closed during this period. Following adaptation, the barrier in the starting zone was opened. The zebrafish was then allowed to freely explore the T-maze, aiming to find and remain in the Enriched Chamber (EC) area for 30 s. Zebrafish successfully entering the EC area were rewarded with food after the 6-min test. If a zebrafish failed to find the EC area within the allotted time, a correction procedure was initiated after 6 min. In this procedure, the barrier on the non-EC side of the T-maze was closed, and the experiment was repeated, guiding the zebrafish from the starting zone to the EC area and encouraging it to stay for 3 min, followed by a food reward.

During formal testing, zebrafish initiated from the starting zone and staying in the Enriched Chamber (EC) area for 30 s were considered as having entered the EC area. The testing duration was set at 6 min. Zebrafish swimming trajectories were recorded and represented by different colored lines based on their speed: white lines indicated slow movement (<2 cm/s), green lines indicated moderate movement (2–5 cm/s), and red lines indicated high-speed movement (>5 cm/s). Swimming trajectories were documented, and parameters such as total swimming distance, average swimming speed, latency to enter the EC area, and cumulative time spent in the EC area were quantified. A longer swimming distance and faster swimming speed within the designated time frame indicated higher vitality. A shorter latency to enter the EC area and longer cumulative time spent in the EC area indicated stronger recognition of the EC area, implying enhanced learning and memory capabilities (Benvenutti et al., 2021).

#### 2.4.7 Sample collection of adult zebrafish experiments

After completing the T-maze behavioral test, brain tissue samples were collected. Thirty zebrafish were randomly selected from each group and anesthetized in ice water. Their brains were dissected and placed in cryovials on ice. Liver and muscle samples were also obtained, rapidly frozen in liquid nitrogen, and stored at −80°C. Additionally, 10 zebrafish from each group were anesthetized in ice water, and their heads were swiftly removed on ice. The excised heads were then placed in 1.5 mL tubes and immersed in Bouin’s solution for 24 h for fixation. Subsequently, brain tissue was dissected to prepare for subsequent H&E staining experiments, and gill samples were also collected (Luo et al., 2024).

#### 2.4.8 Detection of glucose consumption, lactate production and the activities of Hexokinase (HK), Phosphofructokinase (PFK), pyruvate Kinase (PK)

15 zebrafish brain tissues were randomly selected from each group, divided into three portions of 0.1 g tissue each. Each portion was homogenized in 1 mL of extraction buffer on ice. The homogenate was then centrifuged at 8000 *g* for 10 min at 4°C. The supernatant was collected and used for analysis. Glucose content, lactate production, and the activity levels of hexokinase (HK), phosphofructokinase (PFK), and pyruvate kinase (PK) were determined using commercially available assay kits following the manufacturer’s instructions (Li et al., 2022).

#### 2.4.9 Tissue pathological analysis

After fixation in Bouin’s solution for 24 h, brain and gills tissues were sliced in the sagittal plane using a paraffin microtome. Following deparaffinization, 1% methylene blue was applied for 25 min at 37°C, followed by 95% ethanol differentiation for 30 s. The tissues were then subjected to gradient dehydration, xylene transparency, covered with coverslips, and sealed with neutral resin. Hematoxylin and eosin (HE) staining was utilized to observe morphological pathological changes in the zebrafish brain and gills tissues (Luo et al., 2024).

### 2.5 Cell experiments

When BV2 cells reached the logarithmic growth phase, they were harvested and plated at a density of 3×10^5^ cells per well in 6-well plates (He et al., 2024). The experimental groups comprised control, model, bergenin, glycolysis inhibitor 2-deoxy-D-glucose (2-DG), and pyruvate dehydrogenase activator diisopropylamine dichloroacetate (DADA), each with 3 replicate wells. The control group received RPMI 1640 medium, while the model, bergenin, 2-DG, and DADA groups were treated with 50 mM glucose and 10 μM Aβ1-42 oligomers for 24 h to establish a BV2 model of glucose metabolism disorder and inflammation activation. After establishing the High-glucose-induced BV2 model, the bergenin group received continuous administration of 40 μg/mL bergenin, the 2-DG group received 0.2 mM 2-DG, and the DADA group received 1 mM DADA for an additional 24 h. Bergenin was dissolved in DMSO, while 2-DG and DADA were dissolved in sterile water.

### 2.6 Rat experiment

#### 2.6.1 Animal modeling and grouping

After 1 week of acclimation feeding, fifty rats were randomly assigned to five groups: Control (sham surgery), Model, Low-dose (20 mg/kg/d) Bergenin, High-dose (80 mg/kg/d) Bergenin, and Metformin (150 mg/kg/d), each comprising 10 rats. Prior to modeling, rats underwent a 12-h fast, followed by anesthesia with 3% pentobarbital sodium. Using the sixth edition of the “Rat Brain Stereotaxic Atlas” as a guide, their heads were secured in a brain fixation device, with needle insertion coordinates determined relative to the anterior fontanelle: 0.8 mm posteriorly, 1.5 mm laterally to the left and right of the skull midline, and a depth of 3.7 mm from the skull surface (He et al., 2023). In groups other than the sham surgery group, rats received controlled injections of 5 μL STZ (2.4 mg/kg) into each side of the lateral ventricle at a rate of 1 μL/min (Jin et al., 2023). The sham surgery group received 5 μL of normal saline. After injection, the needle remained in place for 5 min before wound closure, and the animals were returned to their cages for observation, awaiting natural awakening. After 2 days post-modeling, intragastric administration was carried out twice daily at 9:00 a.m. and 6:00 p.m. for 14 consecutive days (Sharma et al., 2020). Both the control and model groups received equivalent volumes of normal saline.

#### 2.6.2 Morris water maze

After 14 days of drug intervention, the Morris water maze was conducted. A constant-temperature swimming pool (1.6 m in diameter) was used, and rats were placed into the maze 1 day prior for a 120-s free swim for acclimatization. The maze was divided into four quadrants, and a 12 cm-diameter platform was placed at the center of the first quadrant (platform quadrant) with water added to a depth of 1 cm. Smart 3.0 software recorded the rats’ movement trajectories and time-distance data. Over the first 5 days, rats were placed facing the wall of each of the four quadrants at a fixed time each day, and the software set a 1 min search time for the platform (escape latency). If the rat remained on the platform for more than 2 s within 1 min, the test was terminated, and the time recorded. If not, the escape latency was recorded as 60 s, and the rat was guided to the platform for a 20-s stay for learning and memory. On the 6th day, the platform was removed, and rats were placed into the water from the opposite quadrant of the platform quadrant. The number of times the rats crossed the original platform area within 60 s and the time spent in the platform quadrant were recorded (He et al., 2023).

#### 2.6.3 Sample collection and preservation

The rats were anesthetized with intraperitoneal injections of 3% pentobarbital sodium based on their body weight. Subsequently, their limbs were secured, and a U-shaped incision was made in the abdominal cavity. The right atrium was opened, and 50 mL of pre-cooled physiological saline was injected into the left ventricle until the visceral organs became pale, indicating successful cardiac perfusion (Jin et al., 2023). The head was then severed, and the left brain was fixed in 4% paraformaldehyde. The right brain was divided into the cortex and hippocampus, snap-frozen in liquid nitrogen, and then stored in a −80°C freezer.

#### 2.6.4 Hematoxylin and eosin (HE) staining and Nissl staining

After fixing the left hemisphere, alcohol gradient dehydration, xylene clearing, paraffin embedding, and sectioning using a microtome at a thickness of 3 µm in the coronal plane were performed. Following deparaffinization in xylene and rehydration through a graded series of alcohols, one portion of the sections underwent 5 min staining with hematoxylin, followed by rinsing in running water and differentiation in 0.5% hydrochloric acid ethanol for 1 min, with termination of differentiation by rinsing in running water, immersion in PBS for counterstaining, and final staining with eosin for 1 min. The other portion of the sections underwent 25-min staining with 1% cresyl violet at 37°C, followed by differentiation in 95% ethanol for 30 s and termination of differentiation by rinsing in running water (He et al., 2023). Dehydration was then performed in a gradient of 70%–90% ethanol for 10 min each, followed by absolute ethanol dehydration, xylene clearing, application of neutral resin, covering with coverslips, and air-drying at room temperature. The dorsal hippocampal area was observed under an optical microscope, and photomicrographs were taken for histological analysis.

#### 2.6.5 Detection of glucose consumption, lactate production and the activities of Hexokinase (HK), Phosphofructokinase (PFK), pyruvate Kinase (PK)

Brain tissue samples from nine rats were randomly selected from each group, divided into three portions of 0.1 g tissue each. Each portion was homogenized in 1 mL of extraction buffer on ice. The homogenate was then centrifuged at 8000 *g* for 10 min at 4°C. The supernatant was collected and used for analysis. Glucose content, lactate production, and the activity levels of hexokinase (HK), phosphofructokinase (PFK), and pyruvate kinase (PK) were determined using commercially available assay kits following the manufacturer’s instructions (Pancera et al., 2006).

### 2.7 Quantitative real-time PCR (RT-qPCR)

Total RNA was extracted using the TRIzol method, followed by reverse transcription into cDNA. SYBR Green dye method was utilized with the Bio-Rad CFX96 real-time PCR system for amplification. The PCR program entailed an initial denaturation at 95°C for 10 min, followed by 40 cycles of denaturation at 95°C for 5 s and annealing/extension at 58°C for 30 s (Jin et al., 2023). Melting curve analysis was performed from 65°C to 95°C with 0.5°C increments to detect fluorescence signals. β-actin served as an internal reference, and relative expression levels of target genes were analyzed using the 2^−ΔΔCt^ method. Primer sequences are provided in Table 1.

**TABLE 1:** Primer sequences of PCR.

Gene (zebrafish)	Forward primer (5′-3′)	Reverse primer (5′-3′)
*il1b*	GCT​GCT​GTT​CTT​CAG​GAA​GGA​GAC	TCC​ACC​ATC​TGC​GAA​TCT​TCA​TAC​G
*il6*	GTC​TGC​TAC​ACT​GGC​TAC​ACT​CTT​C	CGT​CCA​CAT​CCT​GAA​CTT​CGT​CTC
*tnfa*	CCA​TAA​GAC​CCA​GGG​CAA​TC	GAT​TCA​GAG​TTG​TAT​CCA​CCT​G
*ins*	GGT​CGT​GTC​CAG​TGT​AAG​CA	CAG​GTG​TTT​CTG​GCA​TTG​GC
*irs1*	GGT​GTC​TTT​TCA​ACA​CCG​CC	TCA​AAA​CAA​GCG​CAG​TCA​GC
*irs2a*	AAG​AGT​GCT​TCA​GTC​AGC​CC	CCT​GCT​CAA​TCT​TGT​ACA​GTG​G
*irs2b*	TAT​GAG​AAT​GGC​GAG​TCC​GC	GAA​AAA​GCG​CTT​GTG​TCC​GT
*pparg*	CTC​TCC​GCT​GAT​ATG​GTG​GAC	GGC​AGA​TCT​GGA​CTG​GTA​GC
*rela*	CCT​GGA​CTC​GTG​GGA​GAG​TA	GGT​CTG​ATC​CGT​GAC​AAT​GTG
*actb1*	ACC​ACG​GCC​GAA​AGA​GAA​AT	ATG​TCC​ACG​TCG​CAC​TTC​AT
Gene (Cell)	Forward primer(5′-3′)	Reverse primer(5′-3′)
IL-1β	TCG​CAG​CAG​CAC​ATC​AAC​AAG​AG	TGC​TCA​TGT​CCT​CAT​CCT​GGA​AGG
IL-6	CTC​CCA​ACA​GAC​CTG​TCT​ATA​C	CCA​TTG​CAC​AAC​TCT​TTT​CTC​A
TNF-α	GTC​TCA​GCC​TCT​TCT​CAT​TCC	CTA​CAG​GCT​TGT​CAC​TCG​AA
β-actin	AAG​TGT​GAC​GTT​GAC​ATC​CG	TCT​GCA​TCC​TGT​CAG​CAA​TG
Gene (Rat)	Forward primer(5′-3′)	Reverse primer(5′-3′)
IL-1β	GTG​TAA​AAC​GCA​GCT​CAG​TAA​CA	TCA​GCA​AGC​AGG​AGT​ACG​ATG
IL-6	CAG​AAT​TGC​CAT​TGC​ACA​ATA​GCA	GAC​AGC​CAC​TGC​CTT​CCC​TAC​TT
TNF-α	CCG​CTT​GGT​GGT​TTG​CTA​CGA​C	GGT​CCC​AAC​AAG​GAG​GAG​AAG​TTC
β-actin	GTG​TAA​AAC​GCA​GCT​CAG​TAA​CA	TCA​GCA​AGC​AGG​CGT​ACG​ATG

### 2.8 Western blot

Total cellular proteins were extracted and quantified using the BCA assay. Protein concentrations were adjusted to 2 μg/μL for sample buffer preparation. Electrophoresis was conducted at 80 V for 30 min, followed by an adjustment to 100 V for 90 min. Wet transfer to a membrane was performed at 200 mA for 90 min. The membrane was then incubated with 5% non-fat milk (prepared in TBST) at room temperature for 1 h. Primary antibodies against GLUT1 (1:1000), HK2 (1:1000), PFKFB3 (1:2000), PKM2 (1:2000), PPARγ (1:1000), NF-κB (1:1000), P-NF-κB (1:1000) and β-actin (1:10000) were applied overnight at 4°C, followed by three washes with TBST for 10 min each. Subsequently, the membrane was incubated with goat anti-rabbit secondary antibody (1:8000) at 37°C for 1 h with shaking, followed by three washes with TBST for 10 min each (He et al., 2023). Chemiluminescence imaging was performed using a gel imaging system, and grayscale analysis was conducted using ImageJ software.

### 2.9 Statistical analysis

The statistical analysis was performed using GraphPad Prism 8.0. The differences between groups were analyzed using One-way analysis of variance (One Way ANOVA) and Pearson Chi-square test. All data are expressed as the mean ± SD. A probability value of *P*< 0.05 was considered to indicate a statistically significant difference.

## 3 Results

### 3.1 The effect of bergenin on high-glucose-induced Zebrafish

#### 3.1.1 The effect of bergenin on High-Glucose-Induced Zebrafish Larvae

**FIGURE 1:**
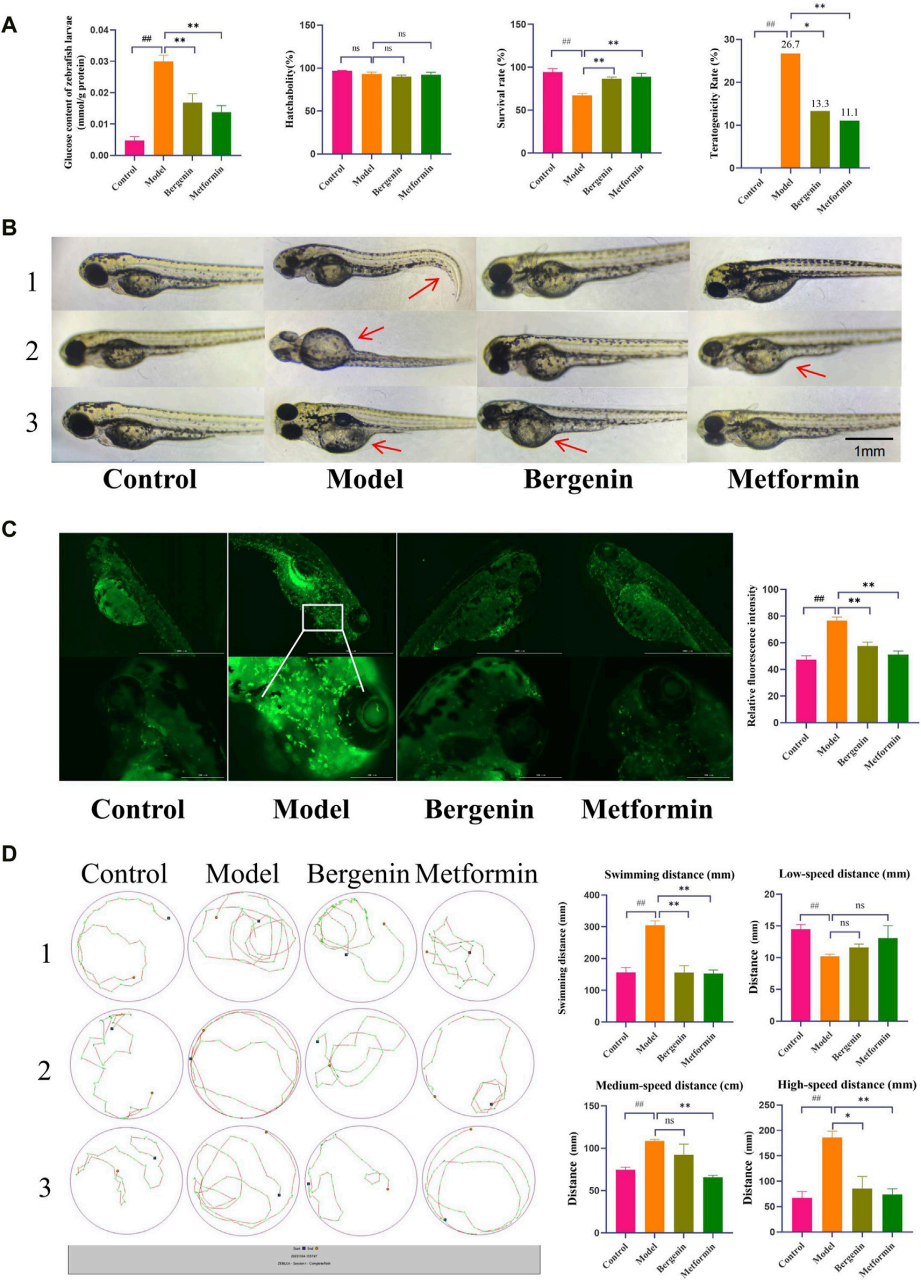
Effects of Bergenin on High-Glucose-Induced Zebrafish Larvae. **(A)** Glucose Content in Zebrafish Larvae; Hatchability, Survival Rate, Teratogenicity Rate of Zebrafish Larvae; **(B)** Teratogenicity in zebrafish larvae; The arrow points to teratogenic effects, such as organ edema or tail malformation; **(C)** Fluorescence Expression in the Brains of Zebrafish Larvae and Relative Fluorescence Intensity; **(D)** Behavioral Trajectory Plot of Zebrafish Larvae and Statistical Analysis of Zebrafish Larval Behavior Data: Total Swimming Distance; Low-Speed Distance (<2 mm/s); Medium-Speed Distance (2–10 mm/s); High-Speed Distance (>10 mm/s). “ns” indicates no statistical significance; ^##^ *P* < 0.01 indicates significance compared to the control group; *P < 0.05, **P< 0.01 indicates significance compared to the model group. (One-way ANOVA and Pearson Chi-square test, Mean ± SD, N = 30).

Using a glucose assay kit, the glucose content in each group of zebrafish was measured to investigate the effect of bergenin on glucose levels in zebrafish larvae induced by high glucose. As shown in Figure 1A, the glucose content in zebrafish larvae in the model group was significantly elevated compared to the control group (*P* < 0.01), with a mean of 0.029 mmol/g protein, which was 6–7 times higher than that of the control group (0.004 mmol/g protein). After administration of bergenin (*P* < 0.01) and metformin (*P* < 0.01), the blood glucose levels significantly decreased. This indicates that the high-glucose-induced zebrafish larval model was established, and bergenin has potential hypoglycemic effects. Next, we evaluated the impact of bergenin on the development of zebrafish larvae induced by high glucose. As shown in Figure 1A, the hatching rates of zebrafish larvae in each group were minimally affected. In Figure 1A, exposure to high glucose resulted in a significant decrease in the survival rate of zebrafish larvae (*P* < 0.01). Conversely, the bergenin (*P* < 0.01) and metformin (*P* < 0.01) treatment groups exhibited significantly increased survival rates. Additionally, the model group displayed developmental teratogenicity such as organ edema and spinal curvature, with a teratogenicity rate of 26.7%. However, the teratogenicity rates decreased to 13.3% and 11.1% in the bergenin and metformin groups, respectively (Figures 1A,B) (Table 2).

**TABLE 2:** Deformities in zebrafish larvae.

Developmental toxicity	Teratogenic effects	Control	Model	Bergenin	Metformin	Ʃt	%
Teratogenic effects	Cardiac edema	0	12	7	4	23	6.4
	Tail malformation^a^	0	8	4	5	17	4.7
	Scoliosis	0	6	0	1	7	1.9
	Yolk edema	0	15	8	6	29	8.1
	Growth retardation^b^	0	10	4	2	16	4.4
	Ʃ Teratogenic embryos	0	24	12	10	41	—
	% Teratogenic embryos	0	26.7^##^	13.3*	11.1**	—	—

^a^ Tail malformation occurred when an embryo had a curved, twisted, or hook-like tail.

^b^ Growth retardation was evaluated by comparing treated embryos with control ones (size, development stage). At 72 and 96 hpf, growth retardation was considered when embryos’ size was less than 2.9 and 3.3 mm, respectively.

^##^ *P* < 0.01 compared with Control group; *P < 0.05, **P < 0.01 compared with Model group (Pearson Chi-square test).

Bergenin significantly affects the activation of zebrafish larval microglial cells induced by high glucose. The transgenic zebrafish line CZ98:*Tg(mpeg1:EGFP)* ^*ihb20Tg/+*^ expresses green fluorescent protein driven by the mpeg1 promoter, specifically labeling macrophages. Activated macrophages exhibit strong green fluorescence. As shown in Figure 1C, high glucose induces increased systemic macrophage activation in zebrafish, with significantly elevated microglial activation in the brain, as indicated by the relative fluorescence intensity (*P* < 0.01). Subsequent treatment with bergenin (*P* < 0.01) and metformin (*P* < 0.01) results in a marked decrease in microglial fluorescence expression in the brain.

Additionally, we also observed the effects of bergenin on the behavior of zebrafish larvae induced by high glucose. Elevated blood glucose levels may lead to increased intracellular oxygen pressure through pathways such as mitochondrial dysfunction, reactive oxygen species production, and blood flow alterations (Kashihara et al., 2022). As illustrated in Figure 1D, zebrafish larvae in the model group exhibited significantly enhanced locomotor activity, with total distance traveled and swimming speed notably higher compared to other groups (*P* < 0.01). High-speed swimming (>10 mm/s) was increased (*P* < 0.01), accompanied by abnormal behaviors such as circling near the edge of the well, known as wall-hugging behavior. Following administration of bergenin (*P* < 0.01) and metformin (*P* < 0.01), swimming speed slowed down, primarily manifesting as medium to low-speed movements (2–10 mm/s, <2 mm/s), and swimming trajectories tended towards normalcy.

#### 3.1.2 The Effects of Bergenin on High glucose-induced Zebrafish Adults

**FIGURE 2:**
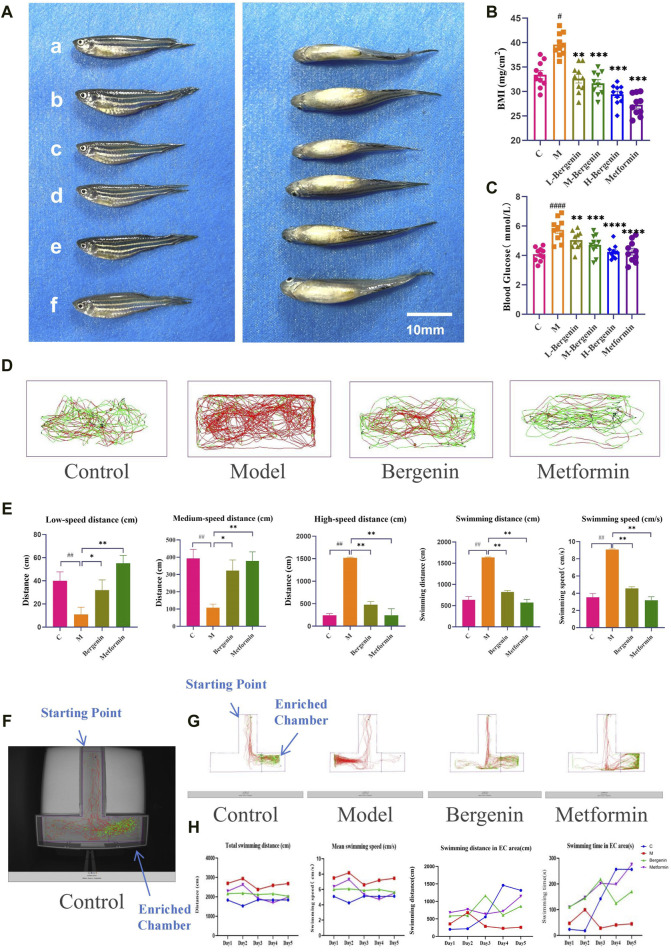
Effects of Bergenin on High-Glucose-Induced Zebrafish Adults. **(A)** Imaging of Adult Zebrafish After Modeling and Drug Administration; **(B)** Body Mass Index (BMI) of Zebrafish Adults; **(C)** Blood Glucose of Zebrafish Adults; **(D)** Zebrafish Behavioral Trajectories; **(E)** Statistical Analysis of Zebrafish Behavior Data: Low-Speed Distance (<2 cm/s); Medium-Speed Distance (2–5 cm/s); High-Speed Distance (>5 cm/s); Total Swimming Distance; Swimming Speed; **(F)** Actual Scene of T-Maze; **(G)** Zebrafish Behavioral Trajectories in the T-maze. **(H)** Statistical Analysis of Zebrafish T-Maze Data: Total Swimming Distance; Mean Swimming Speed; Swimming Distance in EC area; Swimming Time in EC area. ^#^ *P* < 0.05, ^##^ *P* < 0.01, ^####^ *P* < 0.001 indicates significance compared to the control group; *P < 0.05, **P < 0.01, ***P < 0.001, ****P < 0.0001 indicates significance compared to the model group. (One-way ANOVA, Mean ± SD, N = 20).

We first evaluated the effect of bergenin on zebrafish BMI and blood glucose induced by high glucose. As shown in Figures 2A–C, zebrafish in the model group exhibited greater abdominal fat deposition compared to other groups, with a mean BMI of 38.5 mg/cm^2^ and a mean blood glucose level of 5.82 mmol/L, both significantly higher than the control group (*P* < 0.05 and *P* < 0.01). The mean BMI values in the bergenin groups and the metformin group were significantly lower than those in the model group (*P* < 0.01), accompanied by a significant reduction in blood glucose levels compared to the model group (*P* < 0.01). Furthermore, we assessed the impact of bergenin on the behavior of zebrafish induced by high glucose using the adult zebrafish behavioral observation system. As observed in the behavioral swimming trajectories and statistical data presented in Figures 2D,E, zebrafish in the model group predominantly exhibited red-colored swimming trajectories, with significantly higher total distance traveled and swimming speed compared to other groups, along with an increase in high-speed swimming (>5 cm/s) (*P* < 0.01). In contrast, zebrafish treated with varying doses of bergenin and the metformin group showed reduced swimming speed, trending towards more green-colored trajectories, primarily characterized by medium to low-speed movements (2–5 cm/s, <2 cm/s) (*P* < 0.01), indicating a tendency towards normalized swimming trajectories. Finally, we evaluated the effect of bergenin on the learning and memory abilities of zebrafish induced by high glucose through the T-maze experiment. As shown in Figures 2F–H, zebrafish with normal learning and memory abilities can identify food rewards in the EC area through learning. As formal training progresses, the latency to enter the EC area decreases, while the time spent and distance traveled in the EC area increase. Compared to the control group, zebrafish in the model group exhibit higher total distance traveled and average speed, but weaker recognition ability towards the EC area. Interestingly, as learning progresses, the distance and time spent in the EC area decrease. Zebrafish treated with bergenin and metformin show enhanced recognition ability towards the EC area, with significant increases in the time spent and distance traveled in the EC area as training progresses.

#### 3.1.3 The effect of bergenin on inflammatory in high glucose-induced zebrafish

**FIGURE 3:**
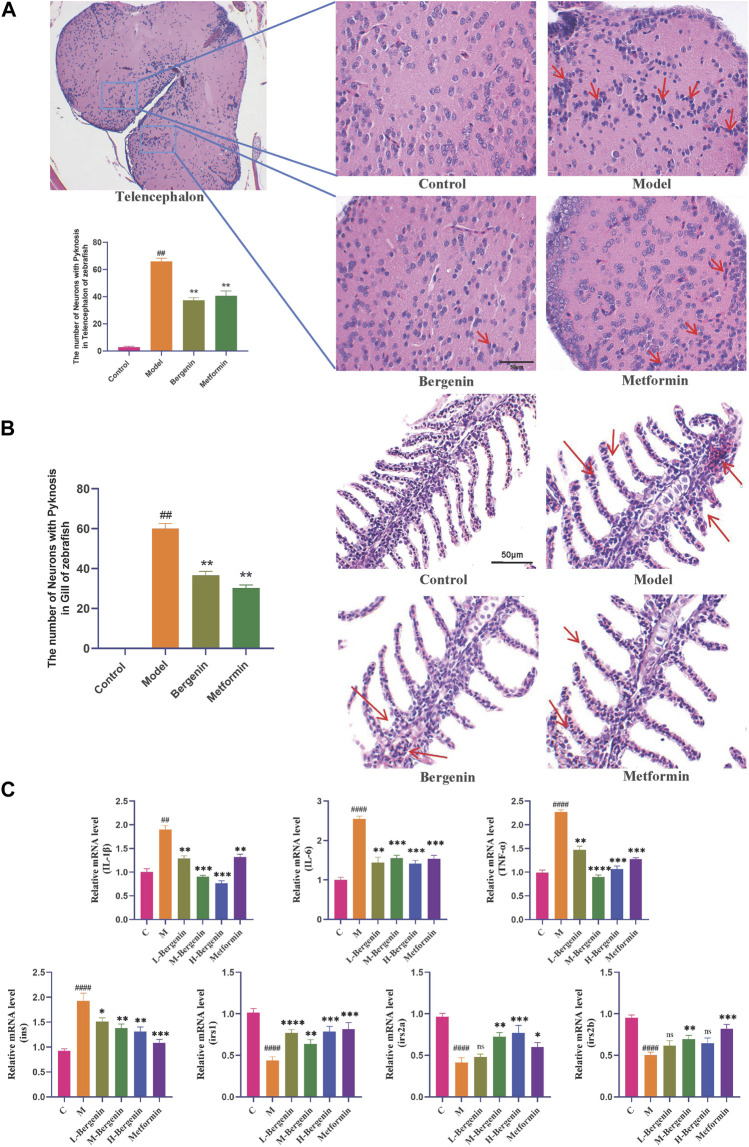
Effects of Bergenin on Inflammation and Insulin Resistance in High-Glucose-Induced Zebrafish Adults. **(A)** Pathological Changes in the Telencephalon Region of High-Glucose-Induced Zebrafish Adults: The arrow indicates activated and aggregated microglial cells (HE staining); **(B)** Pathological Changes in Gill Tissues: The arrow indicates inflammation response in the gill, characterized by capillary disorder, dilation, and congestion (HE staining); **(C)** The Effect of Bergenin on Inflammatory Factors and Insulin Resistance in High Glucose-Induced Zebrafish: Relative mRNA level of *il1b*, *il6*, *tnfa*, *ins*, *irs1*, *irs2a*, and *irs2b*. “ns” indicates no statistical significance; ^##^ *P* < 0.01, ^####^ *P* < 0.0001 indicates significance compared to the control group; *P < 0.05, **P < 0.01, ***P < 0.001, ****P < 0.0001 indicates significance compared to the model group. (One-way ANOVA, Mean ± SD, N = 15).

HE staining revealed an increase in activated microglia, indicating enhanced inflammatory response in the telencephalic region of the model group (*P* < 0.01) (Figure 3A). Gill tissues observations included dilatation of capillaries, capillary disarrangement, vascular congestion, and hyperplasia of epithelial cells on secondary lamellae of the model group (*P* < 0.01) (Figure 3B). After treatment with bergenin and metformin, pathological changes in the telencephalon and gills were partially alleviated, leading to reduced congestion and associated inflammatory alterations (*P* < 0.01).

The impact of bergenin on the expression of inflammatory factors in the brains of high glucose-induced zebrafish was investigated using RT-qPCR. As shown in Figure 3C, compared to the control group, the mRNA levels of inflammatory factors *il1b* (*P* < 0.01), *il6* (*P* < 0.01), and *tnfa* (*P* < 0.01) were significantly increased in the model group. However, the mRNA expression levels of *il1b* (*P* < 0.01), *il6* (*P* < 0.01), and *tnfa* (*P* < 0.01) were significantly decreased in the bergenin and metformin groups compared to the model group, indicating an amelioration in brain inflammation levels.

We investigated bergenin’s impact on the expression of insulin resistance-related genes in the brains of zebrafish induced with high glucose using RT-qPCR. As shown in Figure 3C, compared to the control group, the insulin gene (*ins*) expression was upregulated in the model group, while the expression of insulin receptor substrate genes (*irs1, irs2a, and irs2b*) decreased significantly (*P* < 0.01). However, in both the bergenin and metformin groups (*P* < 0.01), ins expression decreased while *irs1, irs2a, and irs2b* expression increased, indicating bergenin’s effectiveness in alleviating insulin resistance.

#### 3.1.4 The effect of bergenin on glucose, Lactic Acid, and Glycolytic Key Enzymes in High Glucose-Induced zebrafish

**FIGURE 4:**
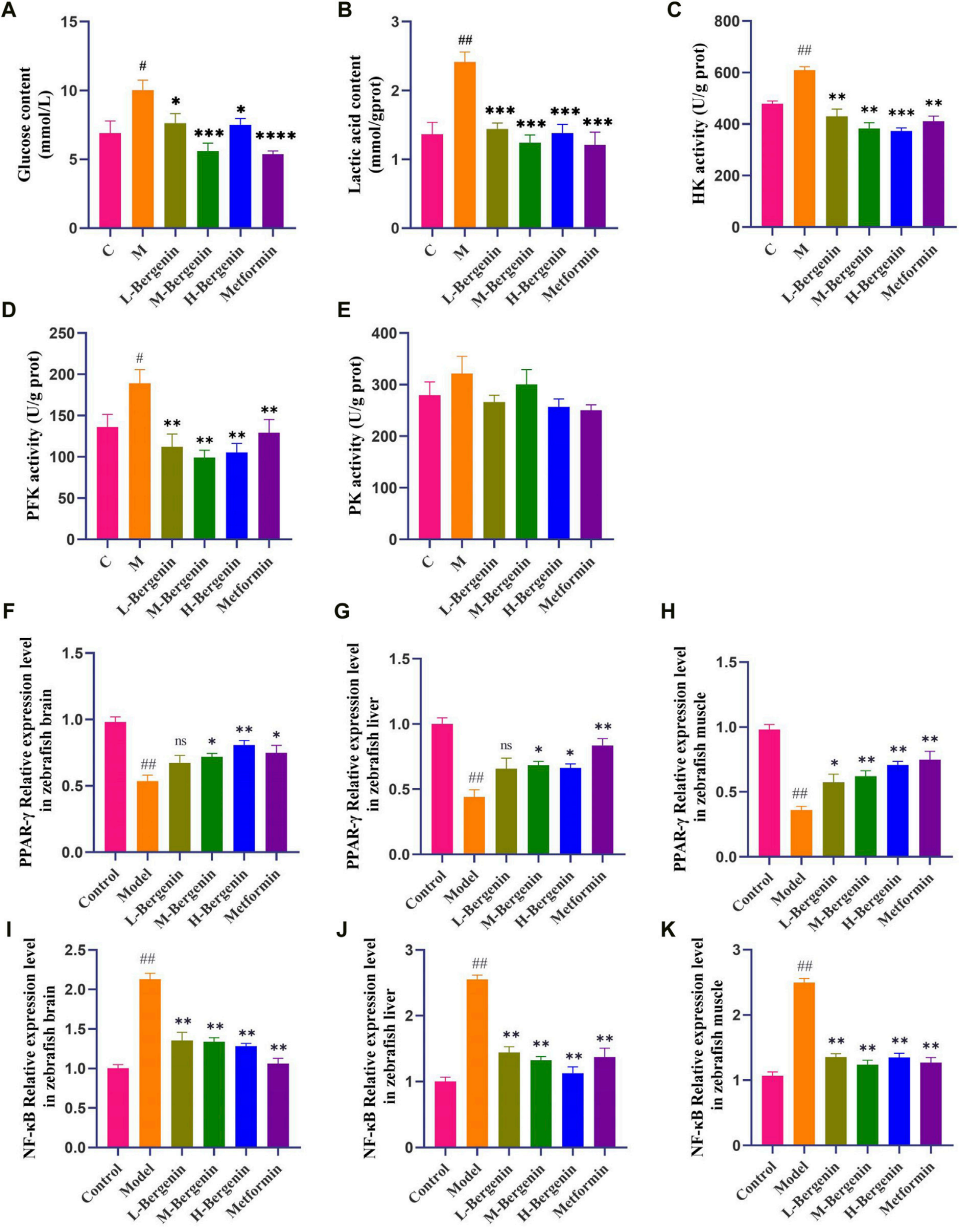
Effects of Bergenin on Glycolysis and PPAR-γ/NF-κB pathway in High-Glucose-Induced Zebrafish Adults. **(A–E)**: The Effect of Bergenin on Glucose, Lactic Acid, and Glycolytic Key Enzymes in High Glucose-Induced Zebrafish: **(A)** Glucose content; **(B)** Lactic acid content; **(C)** HK activity; **(D)** PFK activity; **(E)** PK activity; **(F–K)**: The Effect of Bergenin on PPAR-γ/NF-κB pathway mRNA in High Glucose-Induced Zebrafish: **(F–H)** Relative mRNA levels of *pparg* in the brain, liver, and muscle of zebrafish; **(I–K)** Relative mRNA level of *rela* in the brain, liver and muscle of zebrafish. “ns” indicates no statistical significance; ^#^ *P* < 0.05, ^##^ *P* < 0.01 indicates significance compared to the control group; *P < 0.05, **P < 0.01, ***P < 0.001, ****P < 0.0001 indicates significance compared to the model group. (One-way ANOVA, Mean ± SD, N = 15).

As depicted in Figures 4A–E, zebrafish induced with high glucose exhibited elevated brain glucose levels (*P* < 0.05), increased lactate production (*P* < 0.01), and enhanced activity of glycolytic key enzymes HK and PFK (*P* < 0.05). Subsequent administration of bergenin and metformin resulted in a decrease in brain glucose levels (*P* < 0.05), lactate production (*P* < 0.05), and activity of glycolytic enzymes HK and PFK (*P* < 0.01). These findings indicate disrupted brain glucose metabolism and enhanced glycolysis in zebrafish induced with high glucose. Bergenin and metformin demonstrate the ability to reduce brain glycolysis levels, thereby improving brain glucose metabolism.

#### 3.1.5 The effect of bergenin on PPAR-γ/NF-κB pathway mRNA in high glucose-induced zebrafish

Tissue samples from the brains, livers, and muscles of zebrafish were collected to investigate the impact of bergenin on PPAR-γ/NF-κB pathway-related gene expression induced by high glucose, using RT-qPCR. As depicted in Figures 4F–K, compared to the control group, PPAR-γ expression decreased while NF-κB expression increased in the brains, livers, and muscles of zebrafish in the model group (*P* < 0.01). Conversely, in the bergenin group (*P* < 0.05) and the metformin group (*P* < 0.05), PPAR-γ expression increased while NF-κB expression decreased, suggesting that high glucose induction and bergenin treatment affect multiple tissues in zebrafish. Furthermore, bergenin exerts its neuroprotective effects by activating PPAR-γ and inhibiting NF-κB.

### 3.2 The effect of bergenin on high glucose-induced BV2 cells

#### 3.2.1 The effect of bergenin on inflammatory factors in high glucose-induced BV2 cells

**FIGURE 5:**
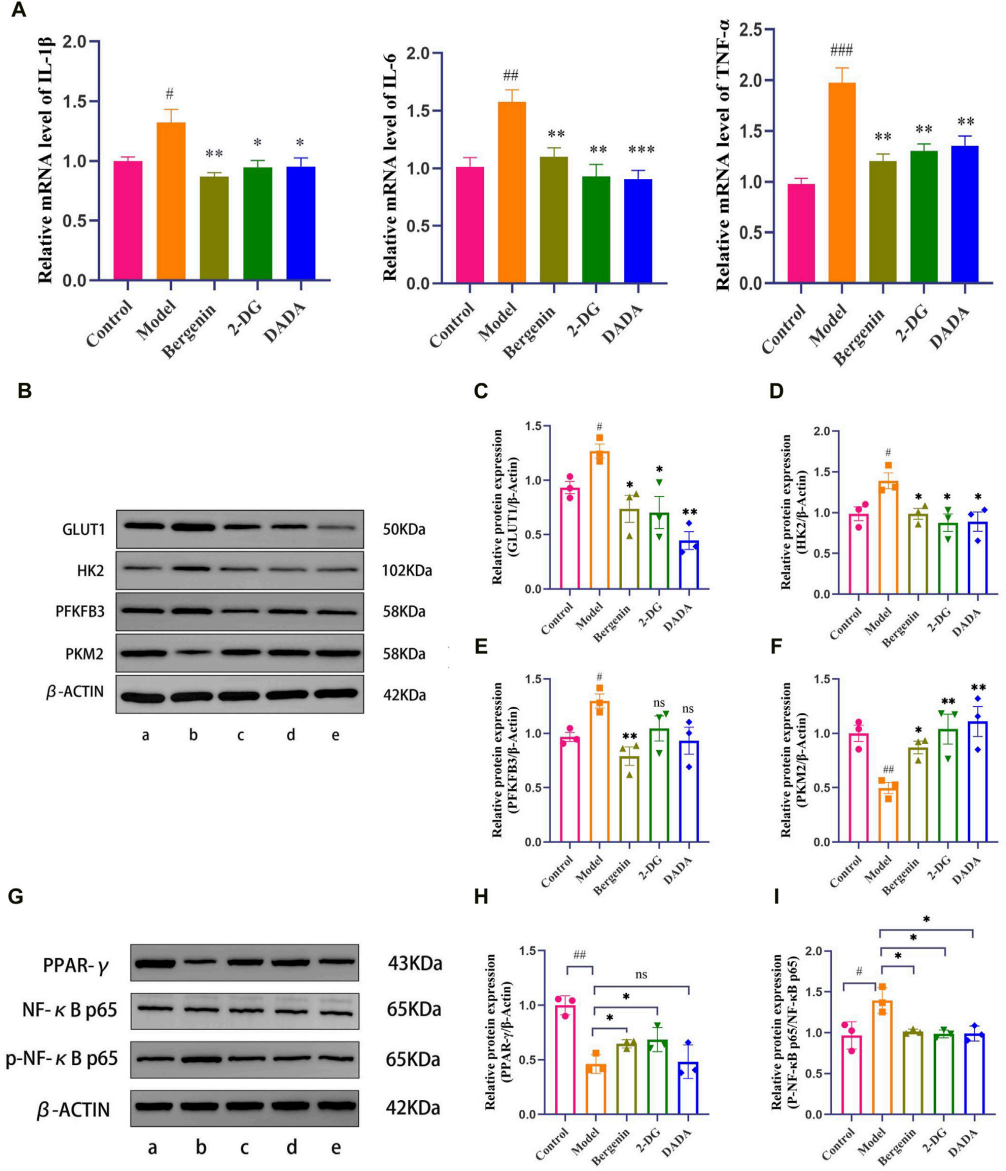
Effects of Bergenin in High Glucose-Induced BV2 Cells. **(A)** The Effect of Bergenin on Inflammatory Factors in High Glucose-Induced BV2 Cells: Relative mRNA level of IL-1β, IL-6, TNF-α; B–F: The Effect of Bergenin on Glycolytic Key Enzymes in High Glucose-Induced BV2 Cells: The Western blot images of glycolysis-related enzymes **(B)**. Data are represented as GLUT1/β-actin **(C)**, HK2/β-actin **(D)**, PFKFB3/β-actin **(E)** and PKM2/β-actin **(F)**. **(G–I)**: The Effect of Bergenin on the PPAR-γ/NF-κB Pathway in High Glucose-Induced BV2 Cells: The Western blot images of PPAR-γ/NF-κB Pathway **(G)**. Data are represented as PPAR-γ/β-actin **(H)** and p-p65/p65 **(I)**. “ns” indicates no statistical significance; ^#^ *P* < 0.05, ^##^ *P* < 0.01, ^###^ *P* < 0.001 indicates significance compared to the control group; *P < 0.05, **P < 0.01, ***P < 0.001 indicates significance compared to the model group. (One-way ANOVA, Mean ± SD, N = 3).

Under bright-field microscopy, changes in cell morphology were observed: BV2 cells in the control group exhibited a regular distribution, without cell aggregation, mostly in a quiescent state, appearing round or spindle-shaped. In comparison, the model group showed a significant increase in amoeboid-like cell clusters. After treatment with Bergenin, 2DG, and DADA on modeled BV2 cells, all three groups exhibited reduced numbers of amoeboid-like cells and fewer clusters compared to the model group. The impact of bergenin on the expression of inflammatory factors in BV2 cell models was studied using RT-qPCR. As shown in Figure 5A, compared to the control group, the model group exhibited increased mRNA expression of inflammatory factors IL-1β (*P* < 0.05), IL-6 (*P* < 0.01), and TNF-α (*P* < 0.01). Following intervention with bergenin, 2DG, and DADA, the mRNA expression of IL-1β (*P* < 0.05), IL-6 (*P* < 0.01), and TNF-α (*P* < 0.01) decreased. This indicates that bergenin, 2DG, and DADA can exert a certain inhibitory effect on inflammation induced in BV2 cells by high glucose through the regulation of the glycolytic pathway.

#### 3.2.2 The effect of bergenin on glycolytic key enzymes in high glucose-induced BV2 cells

High glucose induces metabolic reprogramming, shifting the primary mode of intracellular glucose metabolism from oxidative phosphorylation to aerobic glycolysis. The expression of key enzyme proteins Glucose Transporter Type 1 (GLUT1), HK2, 6-Phosphofructo-2-Kinase/Fructose-2,6-Biphosphatase 3 (PFKFB3), and PKM2 (Pyruvate Kinase M2) during glycolysis was further assessed using Western Blot analysis. As depicted in Figures 5B–F, compared to the control group, the model group showed significantly increased expression of GLUT1, HK2, and PFKFB3 (*P* < 0.05). However, following intervention with bergenin, 2DG, and DADA, the protein expression of GLUT1, HK2, and PFKFB3 in BV2 cells significantly decreased (*P* < 0.05) compared to the model group, indicating that bergenin can exert a glycolysis inhibitory effect similar to that of 2DG, regulating metabolic reprogramming. Additionally, compared to the control group, the expression of PKM2 decreased in the model group. However, following intervention with bergenin, 2DG, and DADA, the expression of PKM2 increased, although the underlying mechanism requires further elucidation.

#### 3.2.3 Bergenin activates PPAR-γ and reduces NF-κB p65 phosphorylation in high glucose-treated BV2 cells

To investigate the protective mechanism of bergenin against high glucose-induced neurotoxicity, we measured the protein levels of PPAR-γ. The results showed a significant decrease in PPAR-γ expression induced by high glucose (Figures 5G,H). This decrease was reversed by bergenin (*P*< 0.05). Concurrently, we assessed the phosphorylation of NF-κB protein, closely associated with cellular inflammatory response. The results indicated a significant increase in p-p65 levels induced by high glucose, which was markedly inhibited by bergenin treatment (*P* < 0.05) (Figures 5G,I). Therefore, we propose that bergenin’s improvement of high glucose-induced glycolysis and increased inflammation is associated with the activation of PPAR-γ and deactivation of its downstream NF-κB pathway.

### 3.3 The effect of bergenin on STZ-Induced rat models

#### 3.3.1 The effect of bergenin on the learning and memory abilities in STZ-induced rat models

**FIGURE 6:**
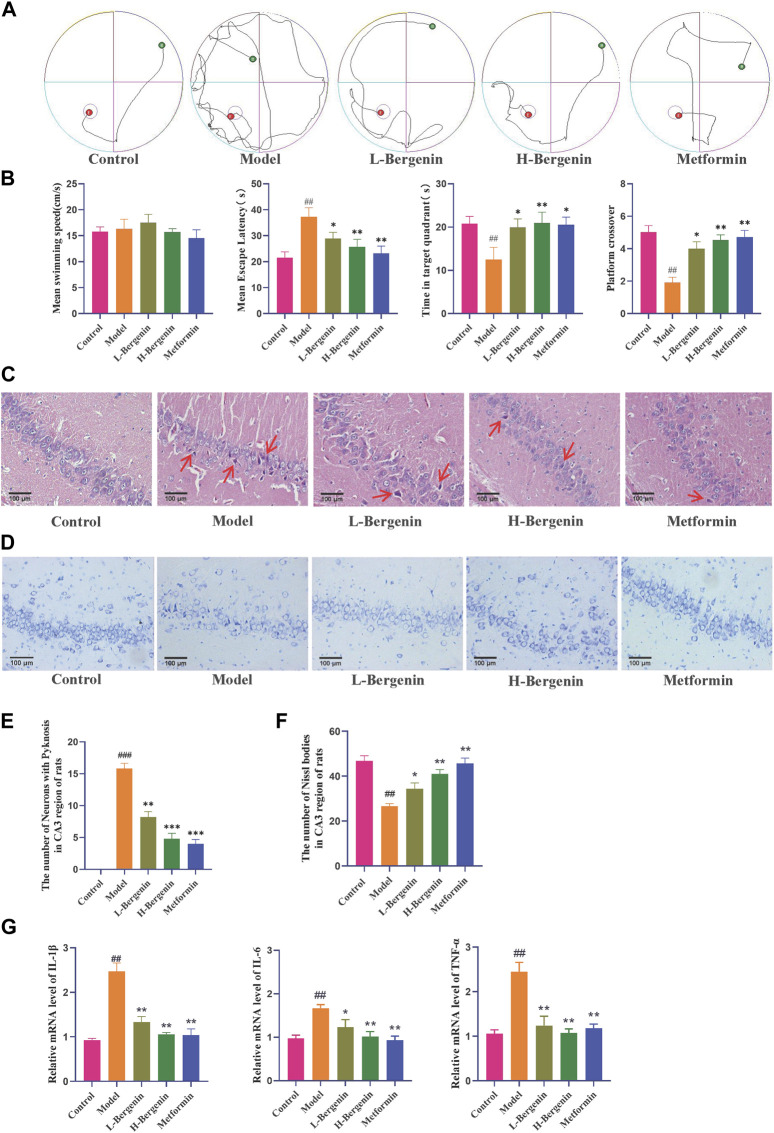
Effects of Bergenin in STZ-Induced Intracerebral Injection Rat Model. **(A)** The Morris Water Maze Trajectory Map evaluated the neurological functions, as well as the learning and memory abilities of rats. **(B)** Statistical data from the Morris Water Maze included the mean swimming speed, latency time to discover the platform, residence time in the quadrant, and frequency of crossing the target platform. **(C–F)**: Effects of Bergenin on the Morphology of Hippocampal Neurons in the STZ-induced Intracerebral Injection Rat Model Detected by HE Staining **(C, E)** and Nissl Staining **(D, F)**, The arrows respectively indicate pyknosis **(C)**; **(G)** The Effect of Bergenin on Inflammatory Factors in STZ-Induced Intracerebral Injection Rat Model: Relative mRNA level of IL-1β, IL-6, TNF-α. ^#^ *P* < 0.05, ^##^ *P* < 0.01 indicates significance compared to the control group; *P< 0.05, **P< 0.01 indicates significance compared to the model group. (One-way ANOVA, Mean ± SD, N = 3).

Compared to the control group, the model group exhibited a decline in learning and memory abilities, with a significantly prolonged time to find the platform (*P* < 0.01). When compared to the model group, the Bergenin low-dose (*P* < 0.05), high-dose (*P* < 0.01), and Metformin (*P* < 0.01) groups showed a reduction in the time to find the platform. After removing the platform, compared to the control group, the model group significantly reduced the time spent in the target platform quadrant (*P* < 0.01) and the number of crossings through the platform (*P* < 0.01). In comparison to the model group, the Bergenin groups exhibited a significant increase in the time spent in the target platform quadrant and an increase in the number of crossings. Bergenin improved the learning and memory abilities in STZ-Induced Rat Model (Figures 6A,B).

HE results showed (Figures 6C,E) that the number of neurons in the CA2 and CA3 regions of rats in the model group decreased, with disordered cell arrangement, nuclear condensation, darkening color, and neuronal damage (*P* < 0.01). Bergenin and Metformin groups showed improved neuronal damage, with normal cell morphology and orderly arrangement. In the low-dose group, there were fewer cells with deepened nuclear staining and pyknosis (*P* < 0.01). Nissl staining results (Figures 6D,F) revealed that in the model group, neurons in the hippocampal region of rats were loosely arranged, with a decrease in Nissl bodies, lightened staining, and cells appearing vacuolated or shrunken, with a reduced number (*P* < 0.01). In the Bergenin and Metformin groups, the number of Nissl bodies inside hippocampal neurons increased, cells were arranged orderly, and cell morphology was normal, with an increased number (*P* < 0.01, *P* < 0.01). Bergenin can reduce abnormal neurons in the hippocampal region of AD rats.

#### 3.3.2 The effect of bergenin on the inflammatory and glycolytic process and in the hippocampus in STZ-induced rat models

Using RT-qPCR to detect the mRNA expression levels of inflammatory factors in the rat hippocampal region, compared with the control group, the model group showed a significant increase in TNF-α, IL-6, and IL-1β mRNA expression levels (*P* < 0.01). Compared with the model group, the expression levels were significantly reduced in the low-dose, high-dose Bergenin, and Metformin groups (*P* < 0.05, *P* < 0.01) (Figure 6G).

**FIGURE 7:**
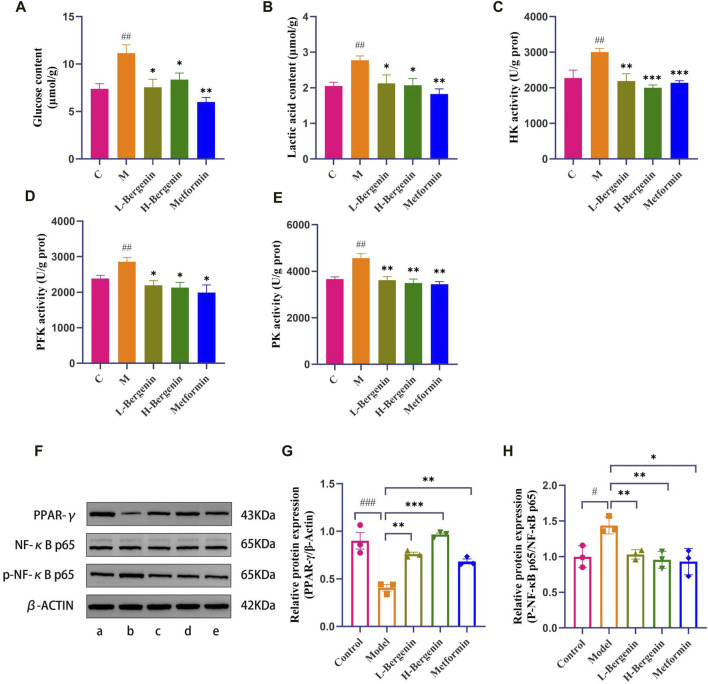
Effects of Bergenin on Glycolysis and PPAR-γ/NF-κB Pathway in STZ-Induced Intracerebral Injection Rat Model. A–E: The Effect of Bergenin on Glucose, Lactic Acid, and Glycolytic Key Enzymes in STZ-Induced Intracerebral Injection Rat Model: **(A)** Glucose content; **(B)** Lactic acid content; **(C)** HK activity; **(D)** PFK activity; **(E)** PK activity; **(F–H)**: The Effect of Bergenin on the PPAR-γ/NF-κB Pathway in STZ-Induced Intracerebral Injection Rat Model: The Western blot images of PPAR-γ/NF-κB Pathway **(F)**. Data are represented as PPAR-γ/β-actin **(G)** and p-p65/p65 **(H)**. ^#^ *P* < 0.05, ^##^ *P* < 0.01, ^###^ *P* < 0.001 indicates significance compared to the control group; *P < 0.05, **P < 0.01,***P < 0.001 indicates significance compared to the model group. (One-way ANOVA, Mean ± SD, N = 3).

As shown in Figures 7A–E, the model group exhibited elevated brain glucose levels (*P* < 0.01), increased lactate production (*P* < 0.01), and enhanced activity of glycolytic key enzymes HK, PFK, and PK (*P* < 0.01). Following administration of bergenin and metformin, there was a significant decrease in brain glucose levels (*P* < 0.05), lactate production (*P* < 0.05), and activity of glycolytic enzymes HK, PFK, and PK (*P* < 0.05). These findings suggest enhanced glycolysis in STZ-induced rat models. Bergenin and metformin demonstrate the ability to reduce brain glycolysis levels, thereby improving brain glucose metabolism.

#### 3.3.3 The effect of bergenin on the PPARγ/NF-κB signaling pathway in the hippocampus in STZ-Induced rat models

WB detection of PPARγ and p-NF-κB p65 protein expression in the hippocampal of rats in each group (Figures 7F–H), the expression of PPARγ in the hippocampus of rats in the model group significantly decreased (*P* < 0.001), while the expression level of p-NF-κB p65/NF-κB p65 significantly increased (*P* < 0.05). In comparison to the model group, the high-dose Bergenin and Metformin groups showed a significant increase in the expression of PPARγ and a significant decrease in the expression of p-NF-κB p65/NF-κB p65 in the hippocampal region.

## 4 Discussion

Diabetes mellitus (DM), a metabolic disorder resulting from lifelong hyperglycemia, is multifactorial. Prolonged hyperglycemia in diabetic patients often leads to widespread chronic damage and dysfunction in various tissues, notably affecting the eyes, kidneys, heart, blood vessels, and nerves, significantly impacting human health (Cloete, 2022). In recent years, there has been increasing attention to DACI. The risk of dementia is reported to be 2.8 times higher in the T2DM population compared to non-T2DM individuals, with an estimated prevalence of mild cognitive impairment (MCI) ranging from 20% to 30% and a dementia incidence of approximately 17.3% among T2DM patients (Zheng et al., 2018; Liang et al., 2023). Clinical studies have shown that T2DM patients commonly exhibit widespread brain atrophy and cerebral microvascular damage, which is age-dependent. Moreover, T2DM-induced reduction in brain volume is three times greater than that of normal aging (Rachdaoui, 2020; Steinbrenner et al., 2022; Miao et al., 2023). High glucose levels have also been identified as a primary cause of excessive phosphorylation of tau protein in hippocampal neurons, contributing to DACI (Chow et al., 2019).

The pathophysiological mechanisms of DACI remain elusive. Evidence suggests that aberrations in brain cell metabolism and heightened levels of inflammatory mediators play significant roles in the onset of DACI (Grandl and Wolfrum, 2018; Shen et al., 2022). Extensive experimental data support the involvement of activated microglial cells in neuronal damage and cognitive impairment in T2DM models (Li et al., 2023). Furthermore, several hypotheses, including insulin resistance, insulin deficiency, disrupted insulin signaling pathways, alterations in brain tissue architecture, changes in cerebral blood flow, immune dysregulation, and mitochondrial dysfunction, have been proposed. These pathophysiological changes further contribute to structural and functional damage to nerve cells, thus affecting cognitive function. Abnormal brain glucose metabolism is closely linked to microglial cell activation, which undergoes metabolic reprogramming from oxidative phosphorylation to glycolysis (Jasleen et al., 2023). Given the significant role of neuroinflammation induced by activated microglial cells in DACI pathogenesis, regulating glucose metabolism to prevent excessive microglial cell activation has emerged as a novel early intervention approach for DACI.

The Traditional Chinese Medicine (TCM) *B. purpurascens* (Hook. f. et Thoms.) Engl., commonly used in China, India, Nepal, and other countries, has been recorded in the “Classification of Herbal Medicines” in China for over a century. Its main active component, bergenin, is recognized for its significant antitussive effect and is included in the *Chinese Pharmacopoeia* as an antitussive and expectorant drug. Modern pharmacological studies have shown that bergenin exerts anti-inflammatory effects by downregulating pro-inflammatory cytokines such as IL-1β, IL-6, and TNF-α(Barai et al., 2018). Furthermore, bergenin exhibits anti-diabetic activity and demonstrates neuroregulatory, acetylcholinesterase inhibitory, antioxidant, and reduction of Aβ_1-42_ and p-*tau* expression levels, supporting its overall neuroprotective effects (Barai et al., 2019; Singla et al., 2022). Our study results indicate that bergenin administration significantly reduces glucose levels, inflammatory pathological manifestations, and levels of inflammatory factors induced by high glucose in zebrafish larvae, adult zebrafish models, and rats injected with STZ into the lateral ventricle. It also improves behavioral abnormalities induced by high glucose and enhances learning and memory abilities. This confirms the dose-dependent hypoglycemic effect and its significant protective effect against inflammation-induced neuronal damage induced by high glucose. The performance characteristics of bergenin highlight its potential as a natural compound for the development of drugs targeting neuronal damage induced by high glucose.

**FIGURE 8:**
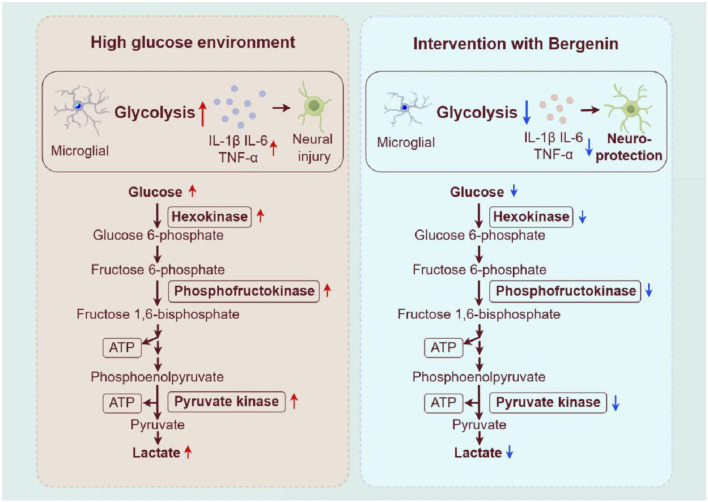
Bergenin regulates glycolysis process to inhibit microglial inflammatory activation. Image created by Figdraw (ID:OAAWI4291b).

Research suggests that peripheral hyperglycemia can enter the brain through active transport, and chronic cerebral hyperglycemia can lead to cerebral hyperinsulinemia, inducing insulin resistance in neurons and increasing the risk of diseases such as DACI and AD (Kellar and Craft, 2020). However, the mechanism by which cerebral hyperglycemia induces neuronal damage remains to be elucidated; some studies suggest that upon developing insulin resistance, a compensatory response may occur, increasing glucose metabolic pathways to meet cellular energy demands, manifested as enhanced glycolysis (Cardoso et al., 2017). Glucose metabolism is a crucial component of energy metabolism, involving oxidative phosphorylation and glycolysis pathways. ATP is generated from glucose breakdown to sustain normal physiological functions. Normally, cerebral glucose metabolism relies on oxidative phosphorylation. However, under pathological conditions, when there’s a sudden demand for ATP, cerebral metabolism shifts to glycolysis due to its faster ATP production rate, despite aerobic conditions (Dienel, 2019). This study reveals that in high-glucose-induced zebrafish and STZ lateral ventricle injection rat models, glucose uptake increases, key glycolytic enzymes are upregulated, and lactate release rises. Similarly, in the high-glucose-induced BV2 cell model, expressions of GLUT-1, HK2, PFKFB3, PKM2 are elevated, but bergenin can modulate glucose metabolism and curb excessive glycolysis (Figure 8).

In the pathological process of neurodegenerative diseases, neuroinflammation mediated by microglia plays a crucial role. Elevated levels of glycolysis may trigger microglial activation, leading to the release of a large number of inflammatory mediators, which adversely affect surrounding neurons (Wu et al., 2023; Yang et al., 2024). Moreover, research suggests that the inflammatory response of microglia can stimulate glycolytic pathway activity while inhibiting oxidative phosphorylation processes (Li et al., 2016). Furthermore, blocking glycolytic metabolism can effectively reduce the immune effects triggered by microglial activation (Lu et al., 2021; Miao et al., 2023). The characteristics of AD and DACI include reduced neuronal glucose uptake, decreased tricarboxylic acid cycle activity, mitochondrial dysfunction, and the interruption of astrocytic energy support to neurons. Additionally, neuroinflammation promotes the competition for glucose by microglia and astrocytes, further exacerbating neuronal glucose hypometabolism. In our study, we observed an increase in glycolytic enzyme activity and expression of inflammatory factors under high glucose conditions in various *in vitro* and *in vivo* models, including zebrafish, cells, and rats. Additionally, increased activation of microglia in the brains of zebrafish and rats was observed. Bergenin was observed to alleviate inflammatory pathological changes induced by high glucose in zebrafish and rats, while also significantly reducing the elevated expression of IL-1β, IL-6, and TNF-α induced by high glucose in zebrafish, BV2 cells, and rats.

**FIGURE 9:**
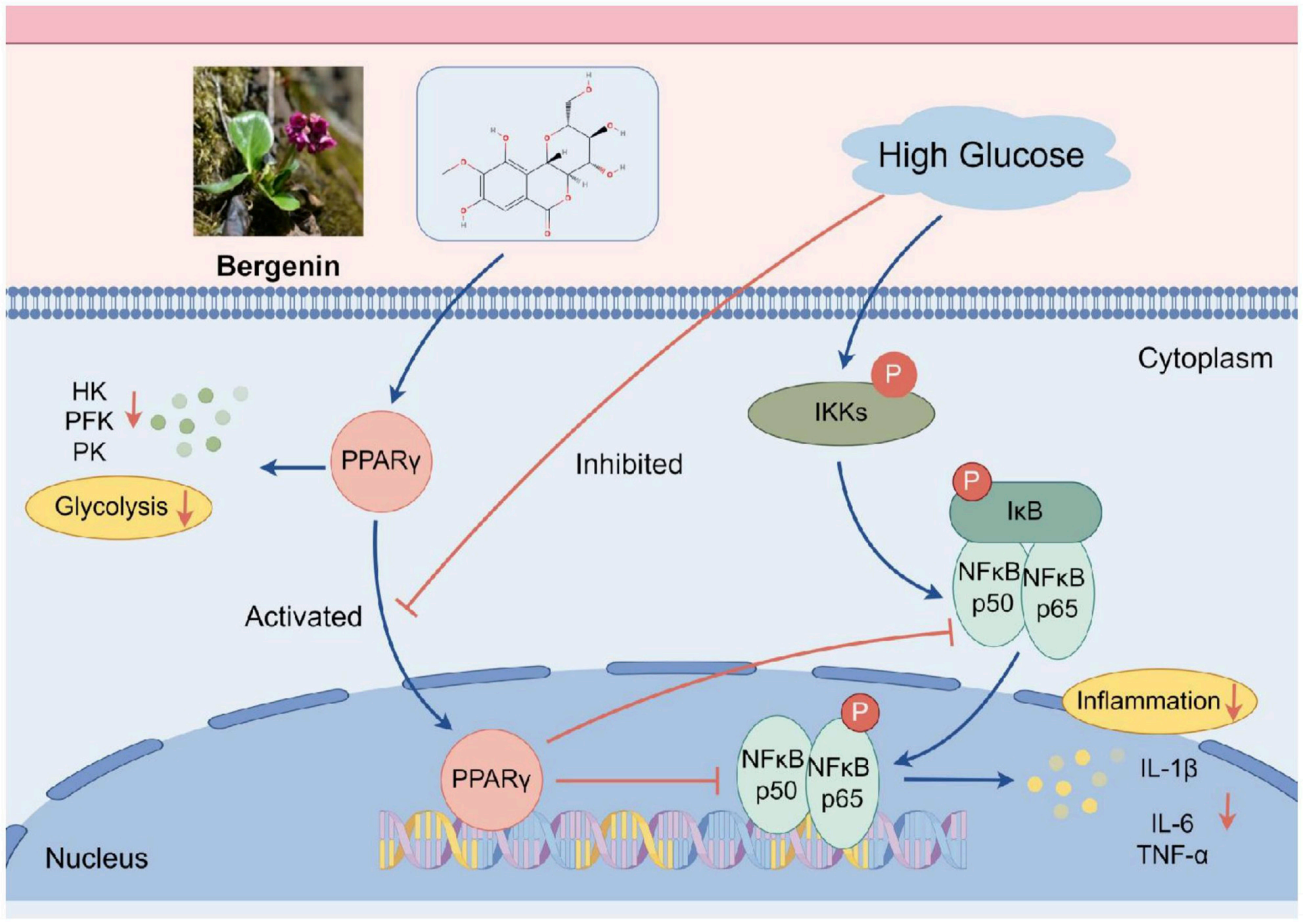
The mechanism of action of bergenin in alleviating inflammation through the PPAR-γ/NF-κB pathway. Image created by Figdraw (ID: UOYRY932b3).

PPARγ, a key nuclear receptor, inhibits the nuclear translocation and DNA-binding activity of NF-κB-p65, thereby reducing the expression of pro-inflammatory cytokines (Luo et al., 2020). Bergenin, an isocoumarin, shows potential as a PPARγ activator. Numerous studies have confirmed Bergenin’s role as a natural PPARγ agonist, inhibiting inflammatory cell aggregation, and being widely used clinically to treat inflammatory-related diseases. NF-κB acts as a key transcription factor in inflammation regulation. Upon activation, NF-κB translocates from the cytosol to the nucleus, where it binds to the promoters of inflammation-related genes, such as IL1-β, IL-6, and TNF-α (Barnabei et al., 2021). Our study demonstrated that bergenin can attenuate the inflammatory stress response of microglial cells by inhibiting glycolytic enzyme activity, thereby exerting neuroprotective effects. Mechanistic investigations revealed that bergenin suppressed the enhancement of glycolysis by activating the PPAR-γ signaling pathway in high-glucose-induced microglial cells. Additionally, bergenin inhibited the NF-κB signaling pathway, reducing the inflammatory response of microglial cells (Figure 9).

The use of multiple models in drug development expands the options available and enables a thorough assessment of drug efficacy. Zebrafish, as a model organism, possess advantages such as small size, large egg production, transparent embryos, and short experimental cycles. In recent years, zebrafish have been increasingly utilized for research on metabolic disorders, brain function mechanisms, and high-throughput drug screening (Gore and Pillay, 2018). The cell model provides more direct cellular-level information, facilitating molecular mechanism studies. Meanwhile, rodent models are more akin to the human physiological environment, capable of simulating complex biological processes and drug metabolism pathways, thus offering crucial insights for clinical translation of drugs. Integrating these models in drug development allows us to capitalize on their respective strengths, enhancing the reliability and effectiveness of research while reducing errors and shortcomings (Gore and Pillay, 2018; Muhammed and Aki-Yalcin, 2019; Barrett et al., 2022). Our study employs a combined approach involving zebrafish, cell, and animal models, providing a fresh perspective and a more thorough evaluation method for drug screening. This approach serves as a significant reference and guide for discovering novel drugs to address diabetes and its related diseases.

While this study provides valuable insights into the protective effects of bergenin against neural damage induced by high glucose, there are several limitations that need to be addressed. Firstly, the experimental models used in this study may not fully recapitulate the complex pathophysiology of neural damage induced by high glucose in humans. Zebrafish, cell, and rat models have their own inherent differences from human physiology, and the findings from these models may not directly translate to clinical applications. Additionally, the mechanisms underlying bergenin’s effects on glucose metabolism and inflammation need further elucidation. Future studies should explore the long-term effects of bergenin treatment, as well as its potential side effects and safety profile.

## 5 Conclusion

This study utilizes zebrafish, cell, and rat models to experimentally demonstrate that high glucose disrupts brain glucose metabolism, triggering inflammatory polarization of microglial cells and subsequent neuronal damage. Bergenin activates PPAR-γ, inhibiting glycolysis enzymes and shifting glucose metabolism from aerobic glycolysis to oxidative phosphorylation. Furthermore, bergenin inhibits IκB phosphorylation and NF-κB activation, reduces the expression of inflammatory factors, and regulates microglial cell activity, thereby alleviating inflammation. In conclusion, bergenin shows potential in mitigating compensatory glycolysis enhancement through the PPAR-γ/NF-κB pathway, alleviate inflammation, and protecting against neural damage induced by high glucose.

## Data Availability

The raw data supporting the conclusions of this article will be made available by the authors, without undue reservation.

## Abbreviations

DACI, Diabetes-associated cognitive impairment; AD, Alzheimer’s disease; 2-DG, 2-Deoxy-D-glucose; DADA, Diisopropylamine dichloroacetate; IL-1β, Interleukin-1β; IL-6, Interleukin-6; TNF-α, Tumor Necrosis Factor-α; GLUT1, Glucose Transporter Type 1; HK2, Hexokinase 2; PFKFB3, 6-Phosphofructo-2-Kinase/Fructose-2,6-Biphosphatase 3; PKM2, Pyruvate Kinase M2; PPAR-γ, Peroxisome Proliferator-Activated Receptor-γ′Abbreviation; NF-κB, Nuclear Factor KappaB.
