# Supplementary material for: Bergenin mitigates neuroinflammatory damage induced by high glucose: insights from Zebrafish, murine microbial cell line, and rat models
Source: Front Pharmacol. 2024 Aug 1;15:1339178. doi: 10.3389/fphar.2024.1339178 (PMC11324488; doi:10.3389/fphar.2024.1339178)
Supplement: Supplementary file 1 [file DataSheet2.PDF]

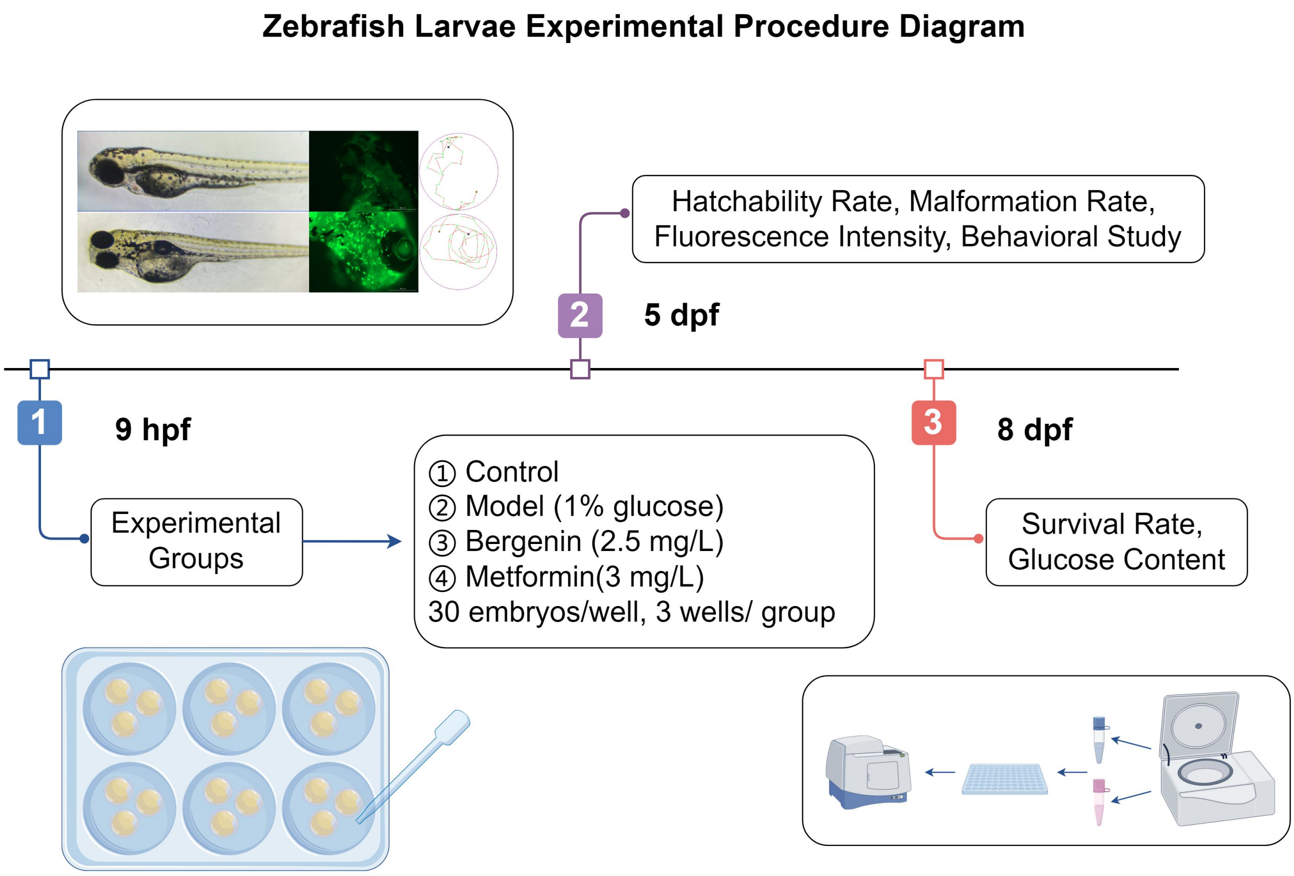
Zebrafish Larvae Experimental Procedure Diagram
Hatchability Rate, Malformation Rate, Fluorescence Intensity, Behavioral Study
2
5 dpf
1
9 hpf
3
8 dpf
Experimental Groups
① Control
② Model (1% glucose)
③ Bergenin (2.5 mg/L)
④ Metformin(3 mg/L)
30 embryos/well, 3 wells/ group
Survival Rate, Glucose Content

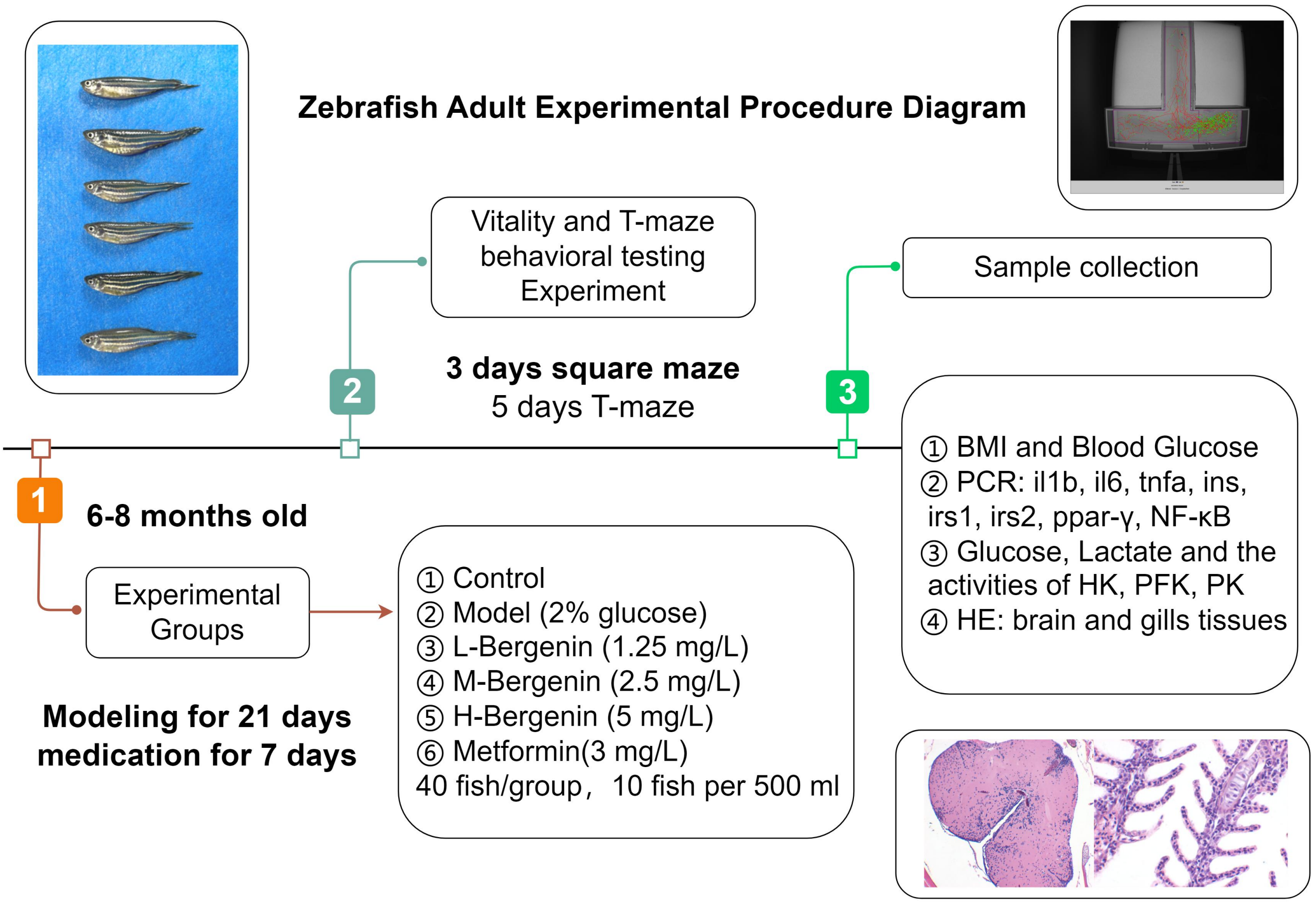
Zebrafish Adult Experimental Procedure Diagram
Vitality and T-maze behavioral testing Experiment
Sample collection
2
3 days square maze
5 days T-maze
3
① BMI and Blood Glucose
② PCR: il1b, il6, tnfa, ins, irs1, irs2, ppar-γ, NF-κB
③ Glucose, Lactate and the activities of HK, PFK, PK
④ HE: brain and gills tissues
1
6-8 months old
Experimental Groups
① Control
② Model (2% glucose)
③ L-Bergenin (1.25 mg/L)
④ M-Bergenin (2.5 mg/L)
⑤ H-Bergenin (5 mg/L)
⑥ Metformin(3 mg/L)
40 fish/group， 10 fish per 500 ml
Modeling for 21 days
medication for 7 days

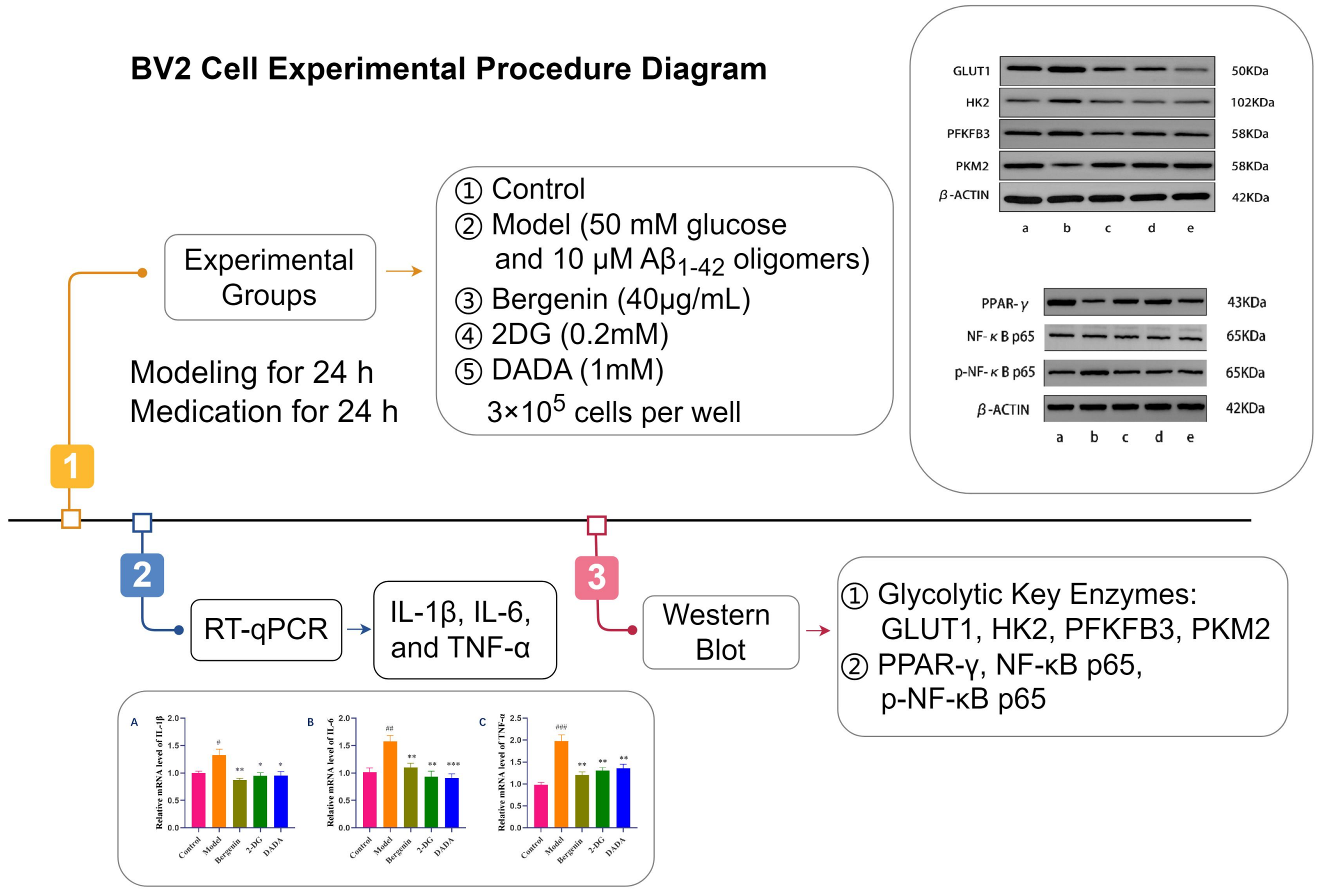
BV2 Cell Experimental Procedure Diagram
1
Experimental Groups
Modeling for 24 h
Medication for 24 h
① Control
② Model (50 mM glucose and 10 μM Aβ1-42 oligomers)
③ Bergenin (40μg/mL)
④ 2DG (0.2mM)
⑤ DADA (1mM)
3×10^5 cells per well
GLUT1 50KDa
HK2 102KDa
PFKFB3 58KDa
PKM2 58KDa
β-ACTIN 42KDa
a b c d e
PPAR-γ 43KDa
NF-κB p65 65KDa
p-NF-κB p65 65KDa
β-ACTIN 42KDa
a b c d e
2
RT-qPCR
IL-1β, IL-6, and TNF-α
3
Western Blot
① Glycolytic Key Enzymes: GLUT1, HK2, PFKFB3, PKM2
② PPAR-γ, NF-κB p65, p-NF-κB p65
Control
Model
Bergenin
2-DG
DADA

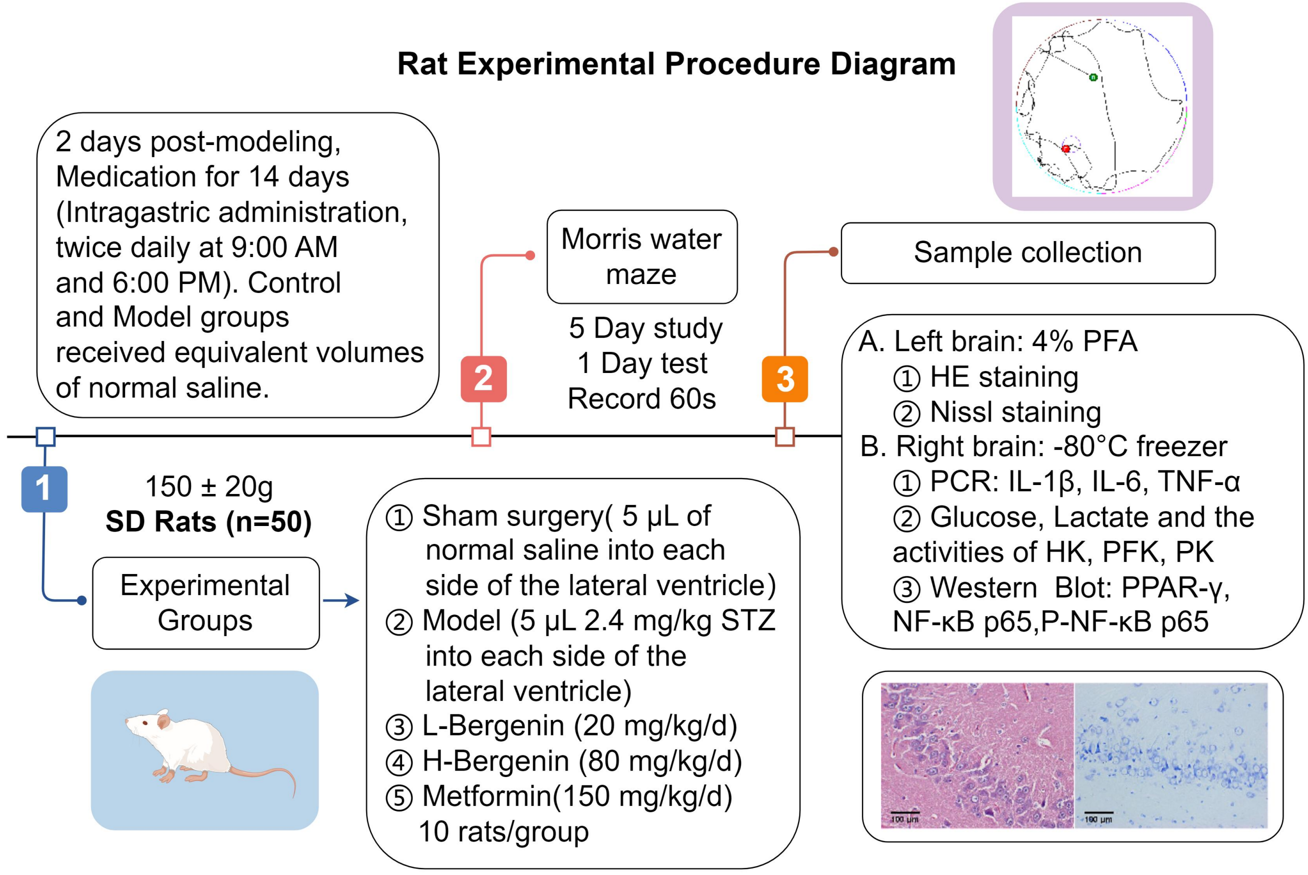
Rat Experimental Procedure Diagram
2 days post-modeling, Medication for 14 days (Intragastric administration, twice daily at 9:00 AM and 6:00 PM). Control and Model groups received equivalent volumes of normal saline.
1
150 ± 20g
SD Rats (n=50)
Experimental Groups
① Sham surgery(5 μL of normal saline into each side of the lateral ventricle)
② Model (5 μL 2.4 mg/kg STZ into each side of the lateral ventricle)
③ L-Bergenin (20 mg/kg/d)
④ H-Bergenin (80 mg/kg/d)
⑤ Metformin(150 mg/kg/d)
10 rats/group
2
Morris water maze
5 Day study
1 Day test
Record 60s
3
Sample collection
A. Left brain: 4% PFA
① HE staining
② Nissl staining
B. Right brain: -80°C freezer
① PCR: IL-1β, IL-6, TNF-α
② Glucose, Lactate and the activities of HK, PFK, PK
③ Western Blot: PPAR-γ, NF-κB p65,P-NF-κB p65
