# Supplementary figures and images for: Bergenin mitigates neuroinflammatory damage induced by high glucose: insights from Zebrafish, murine microbial cell line, and rat models
Source: Front Pharmacol. 2024 Aug 1;15:1339178. doi: 10.3389/fphar.2024.1339178 (PMC11324488; doi:10.3389/fphar.2024.1339178)

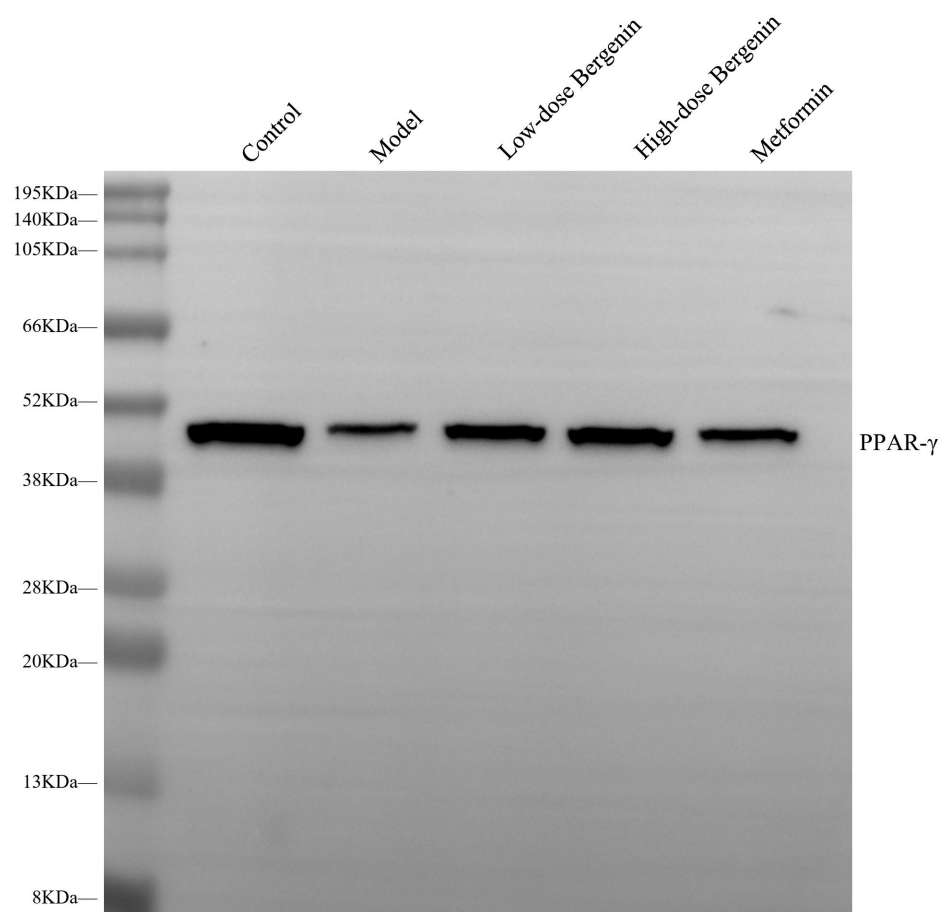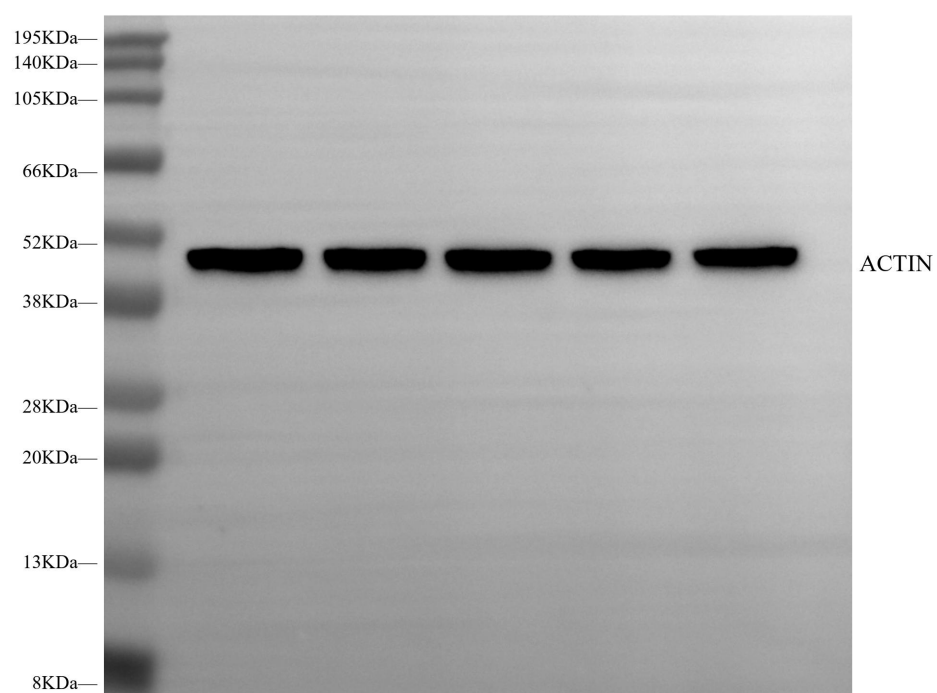

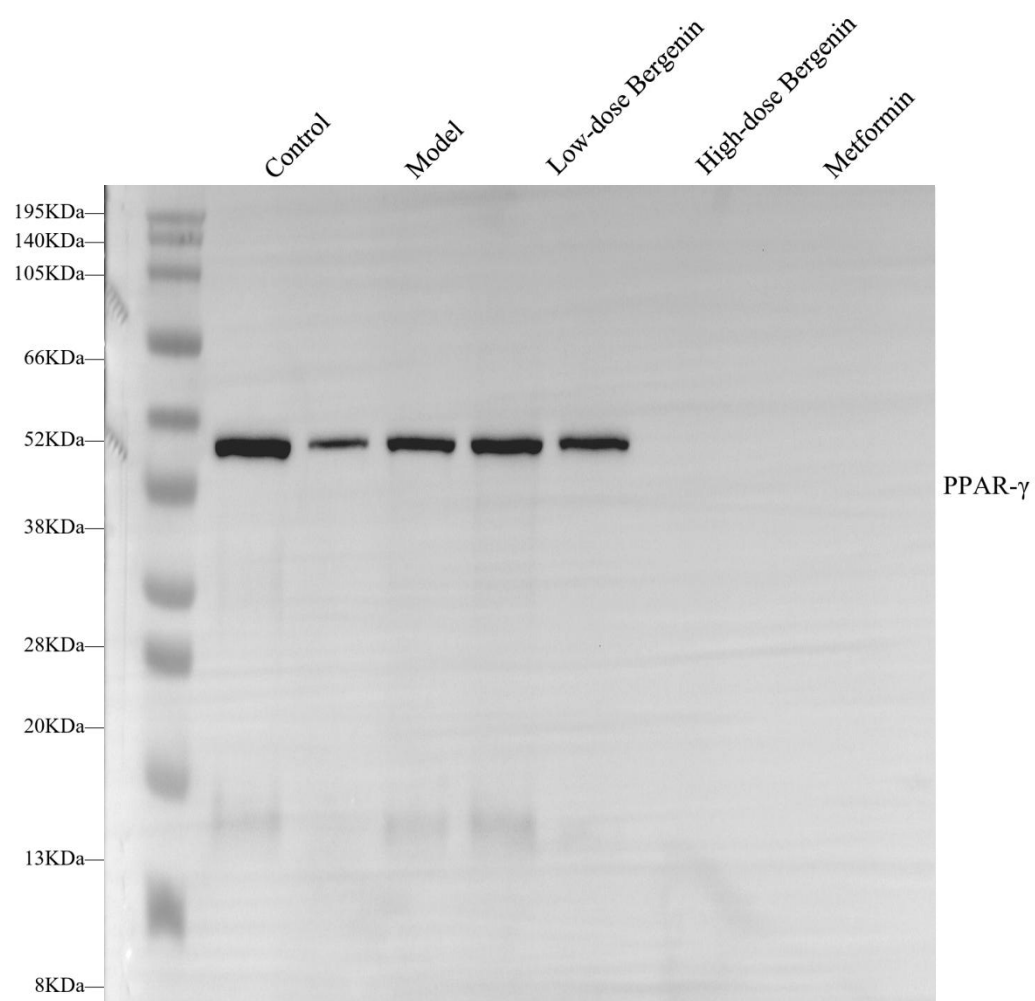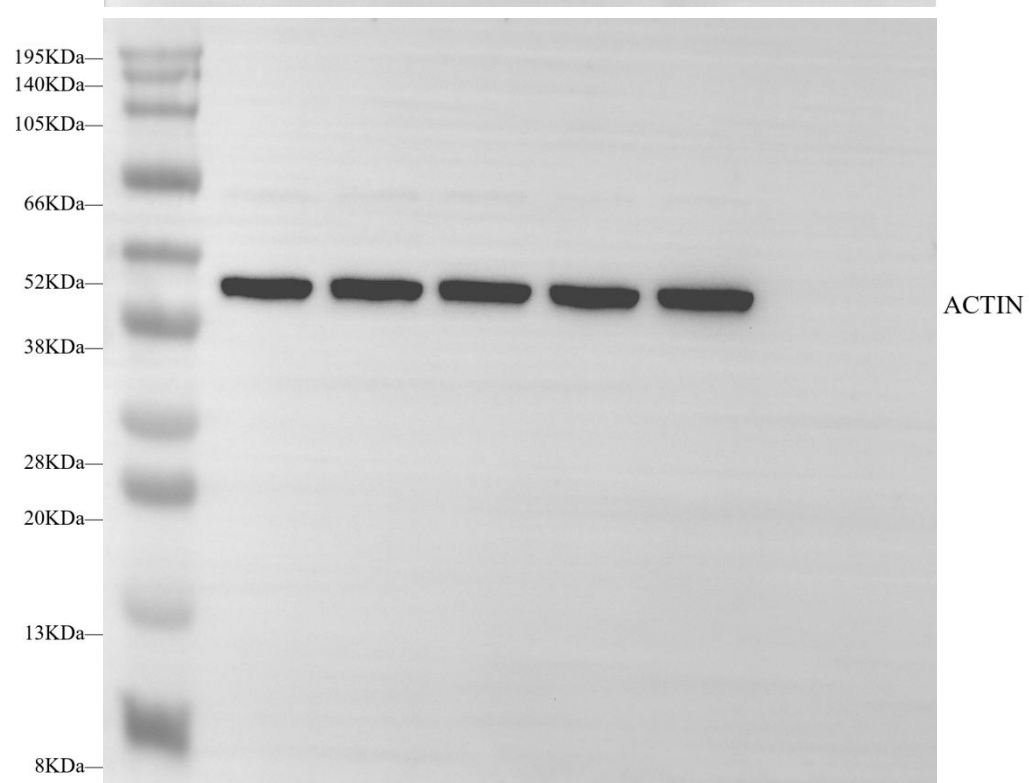

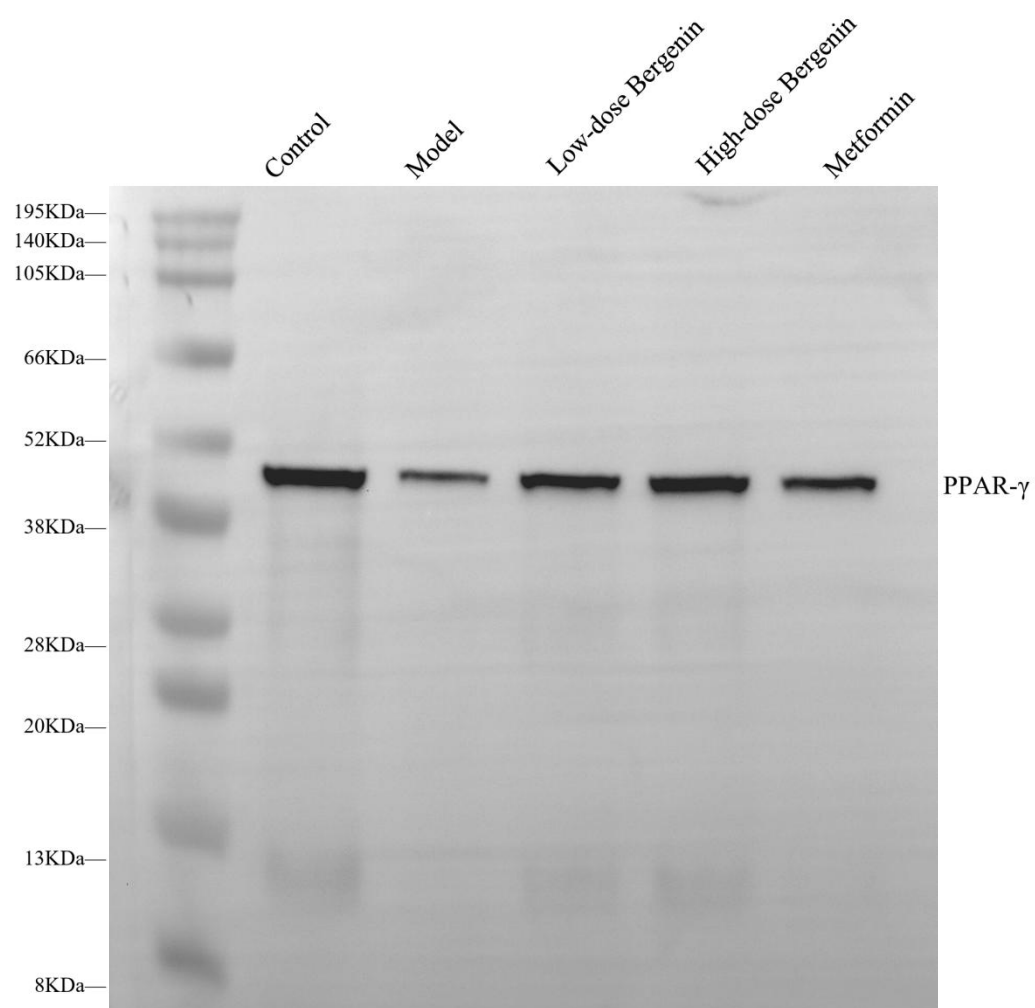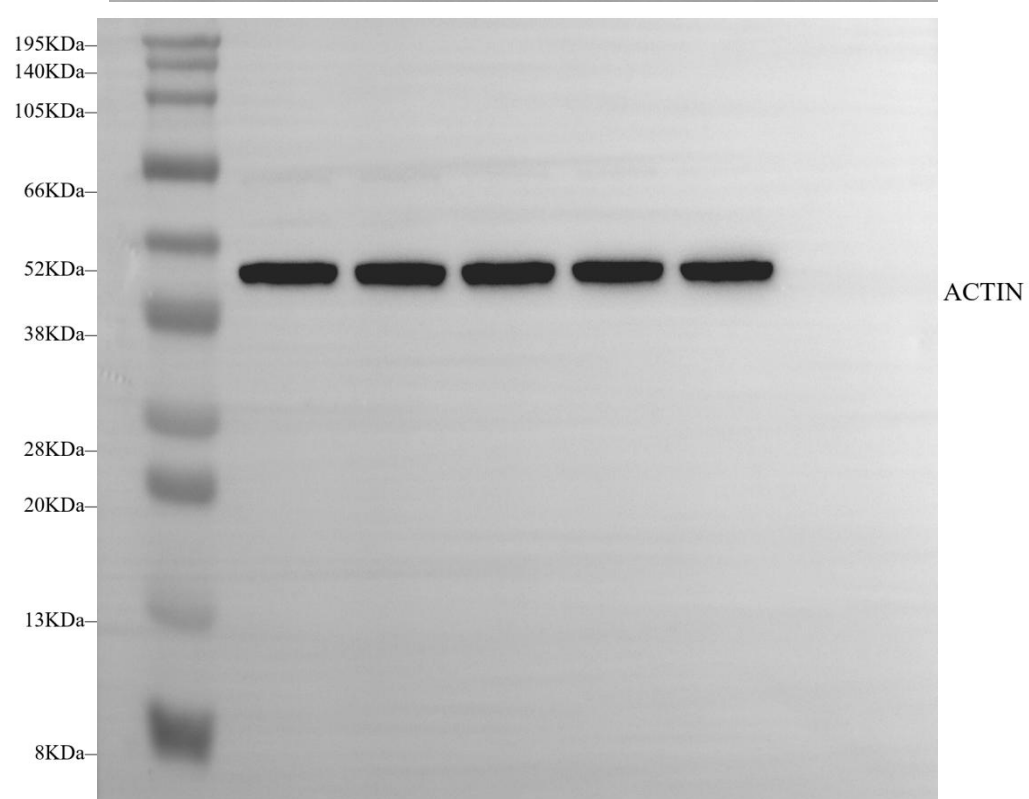

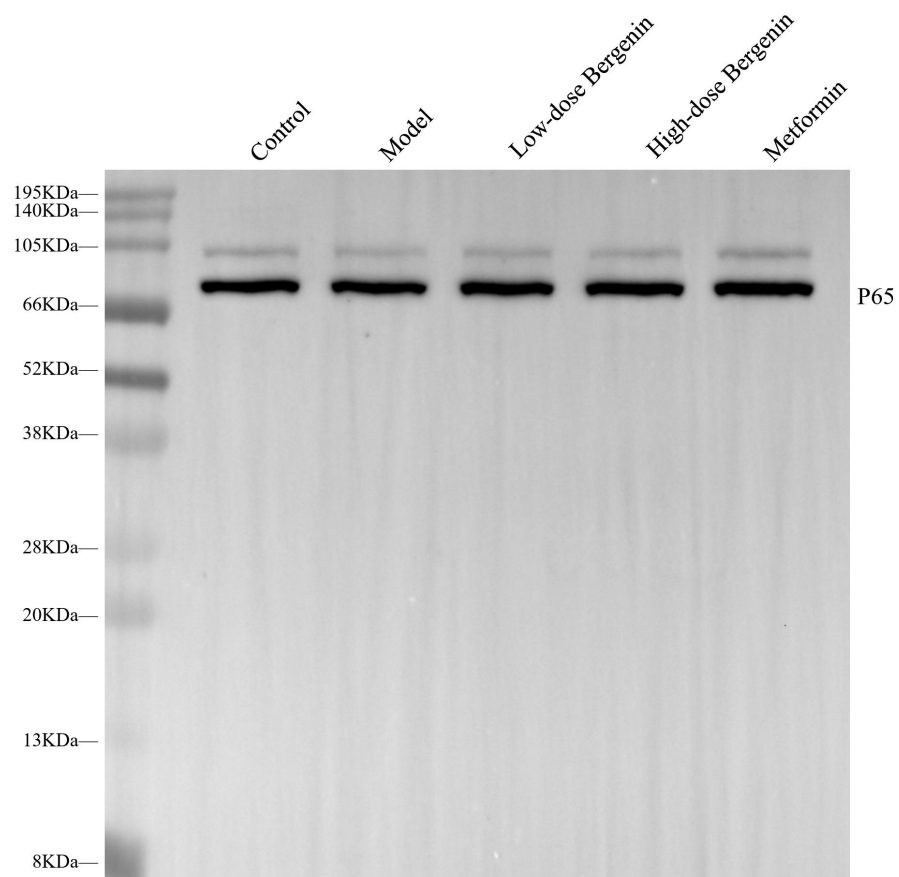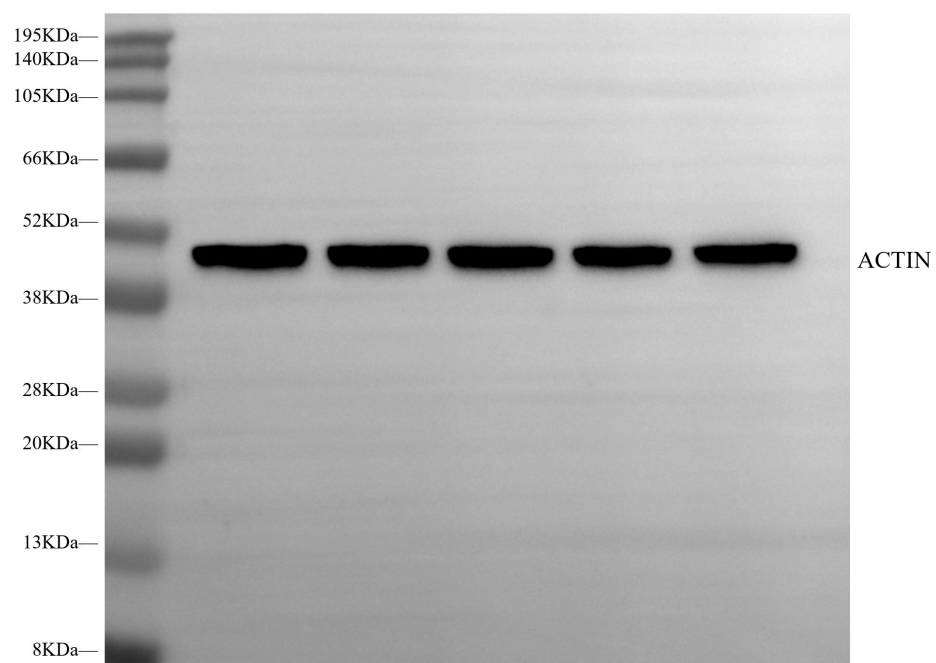

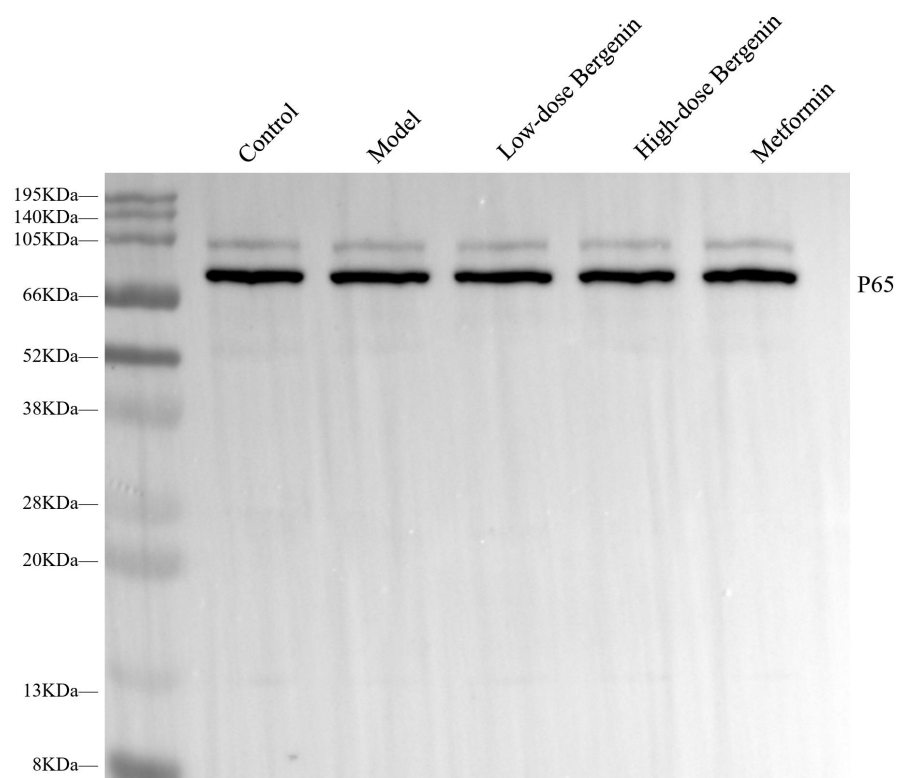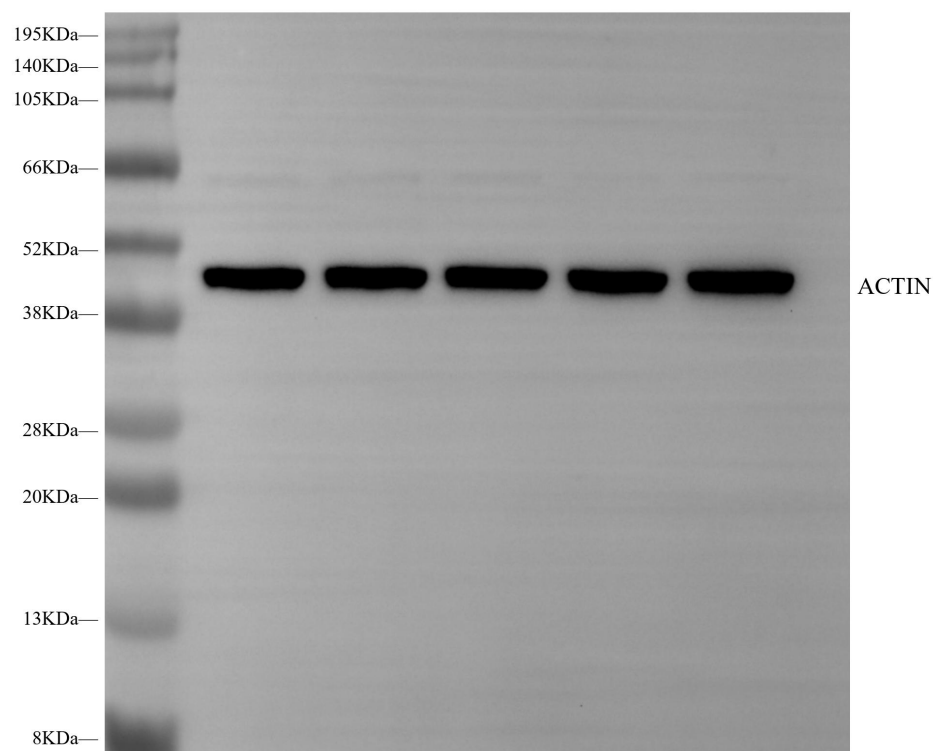

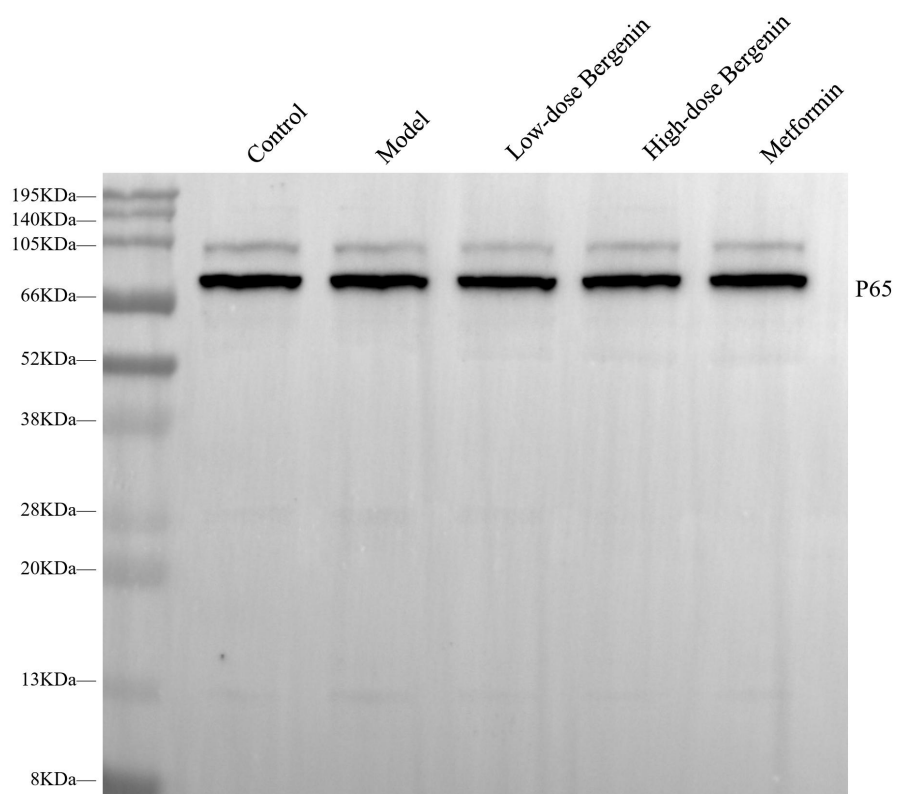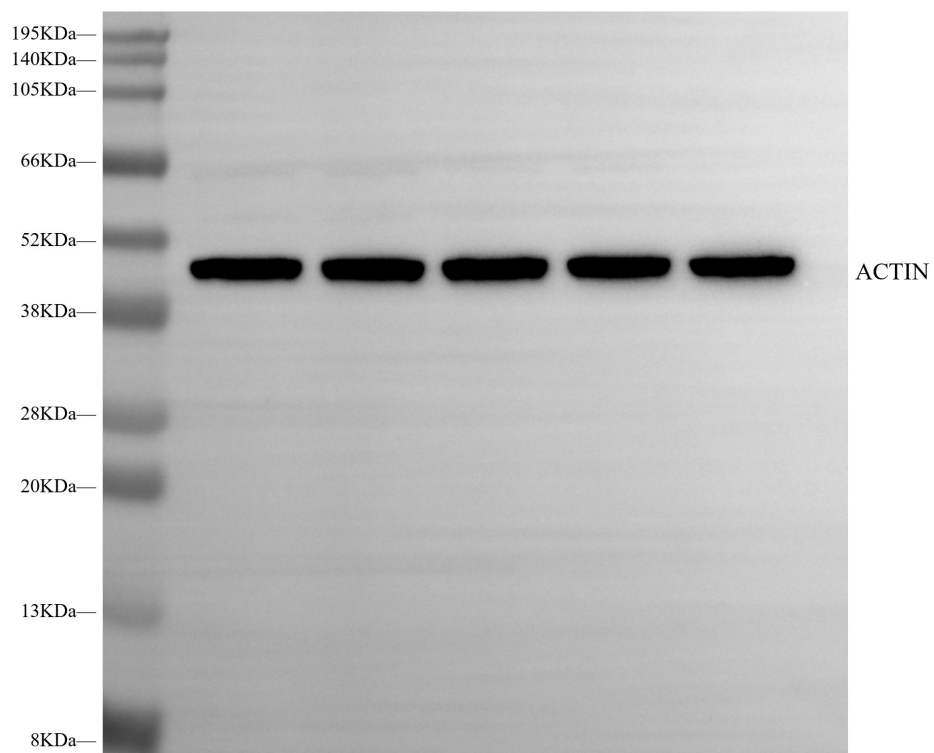

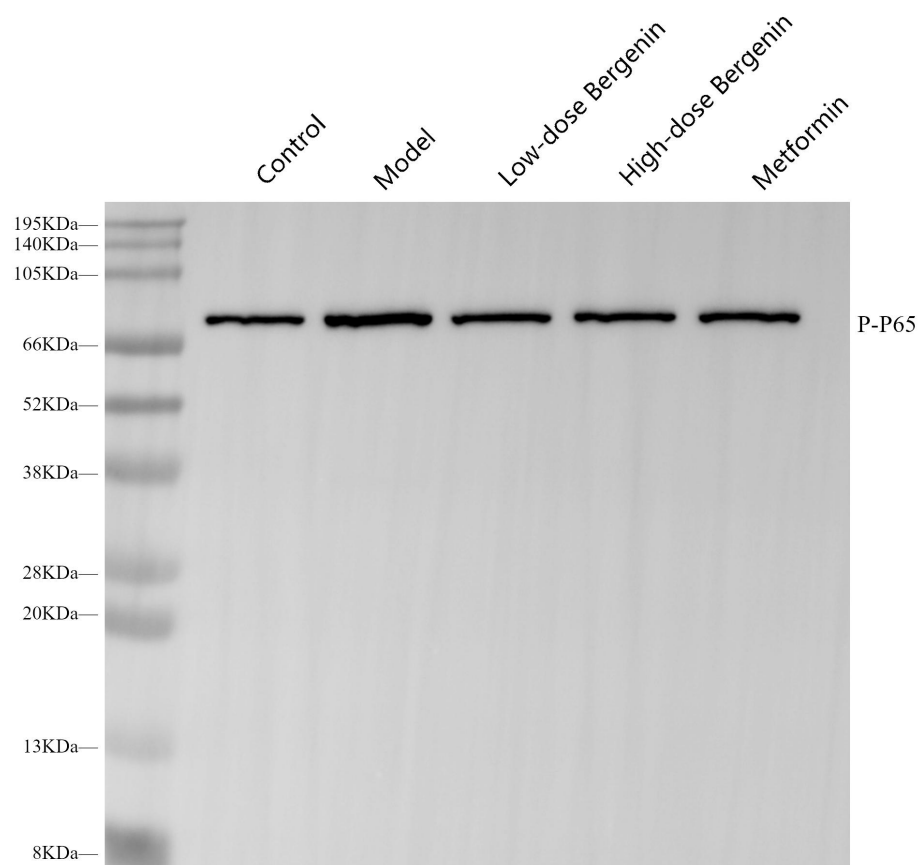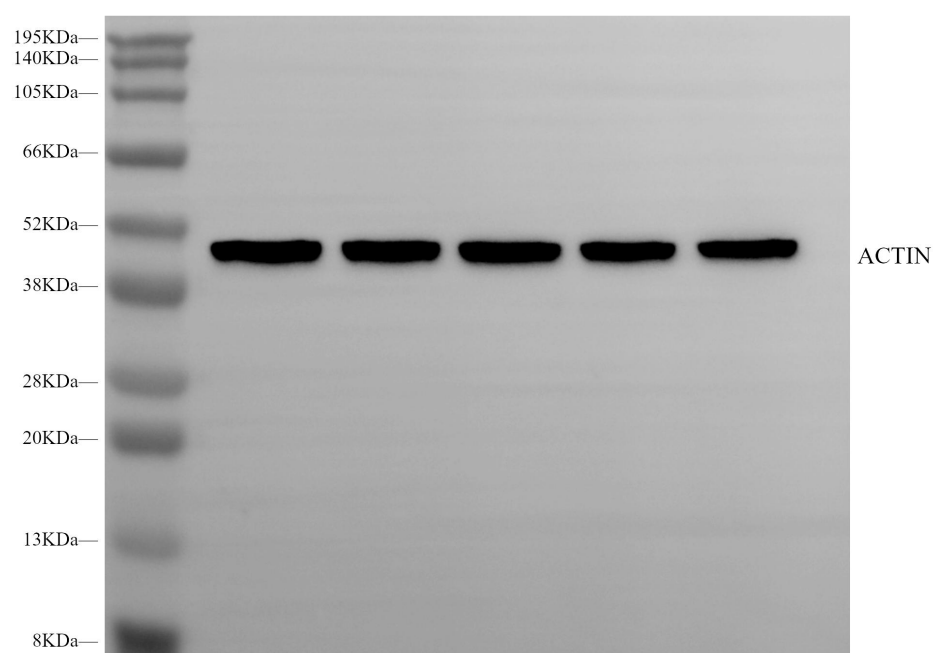

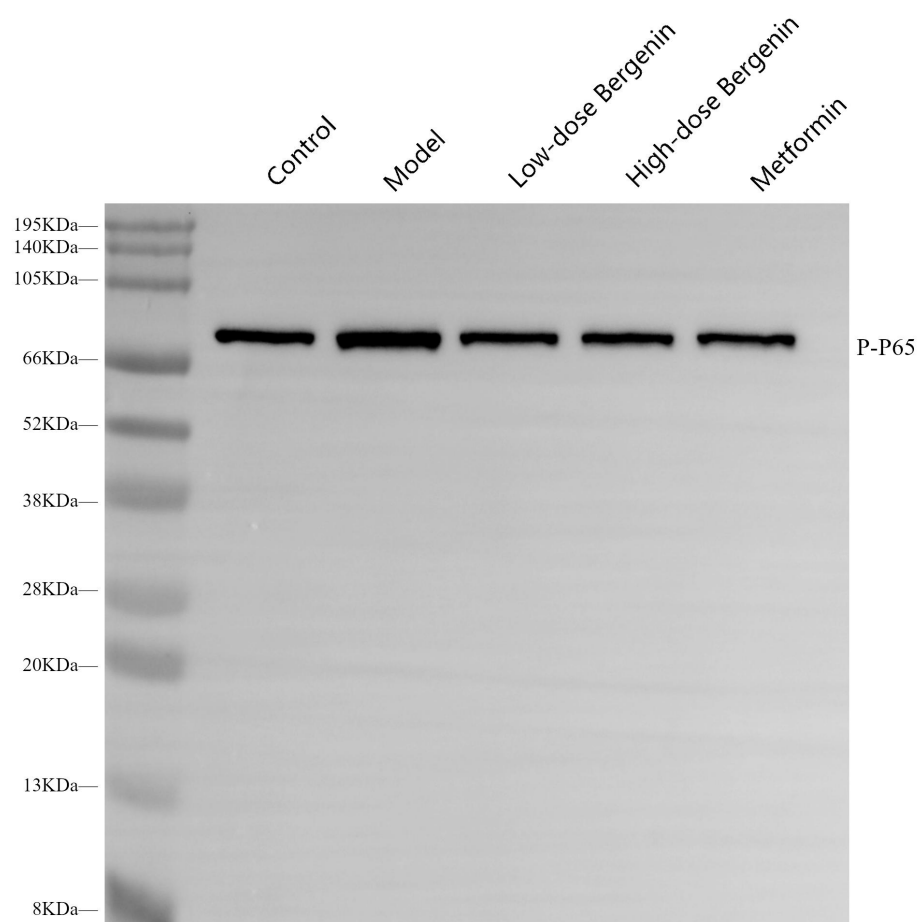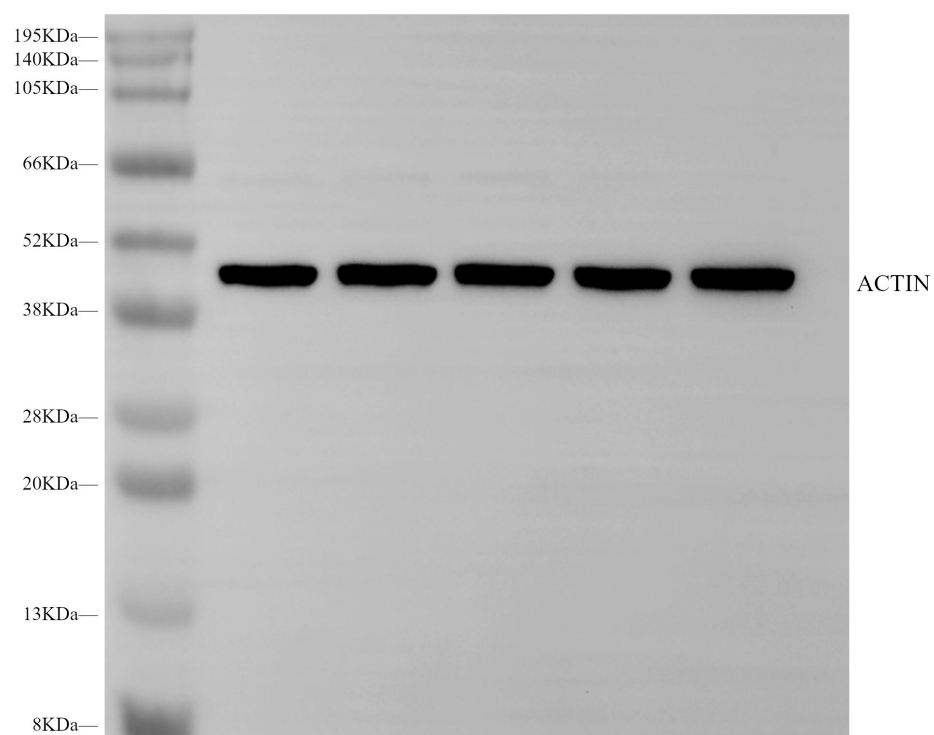

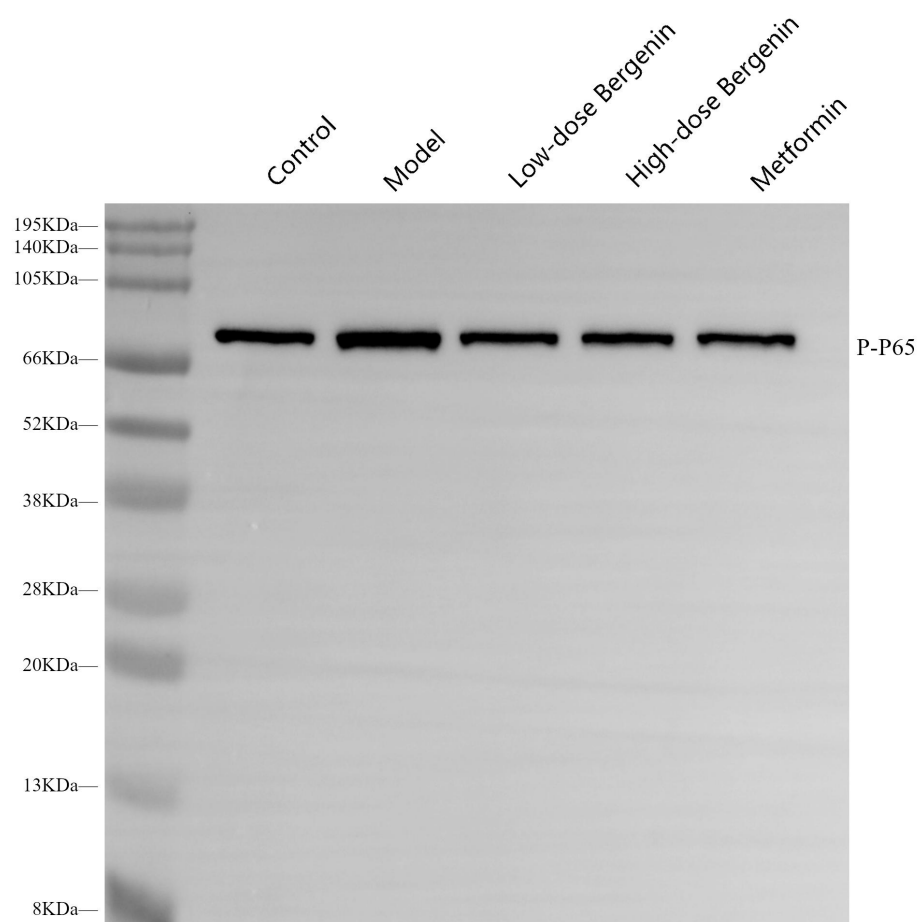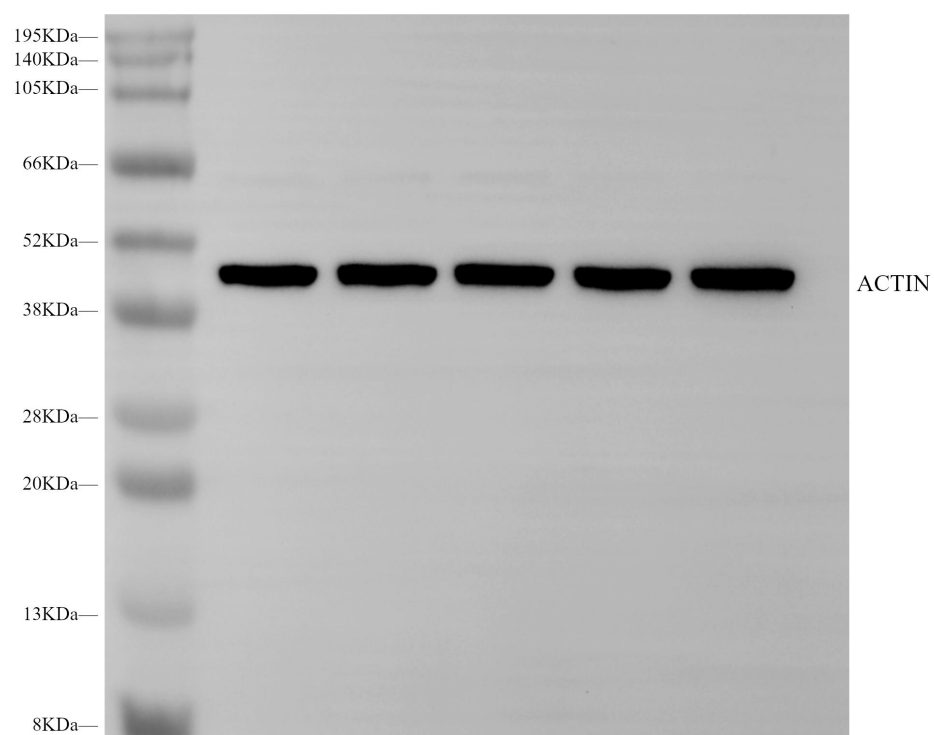

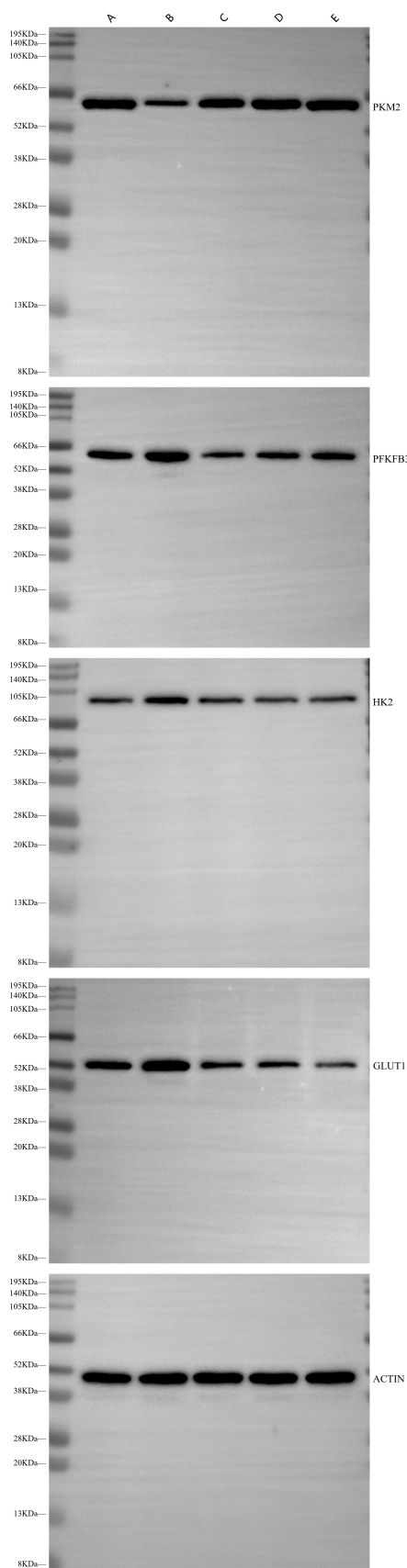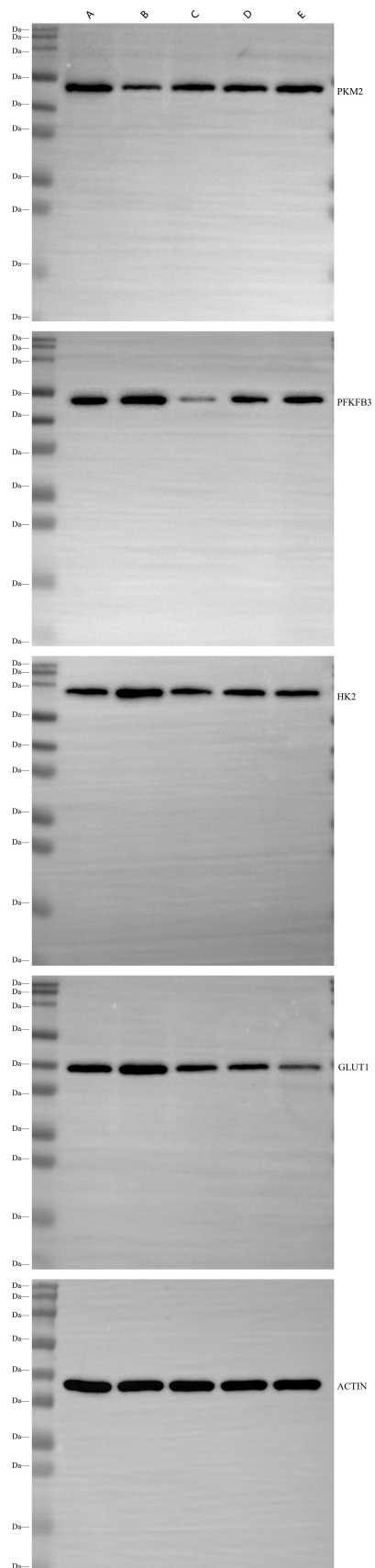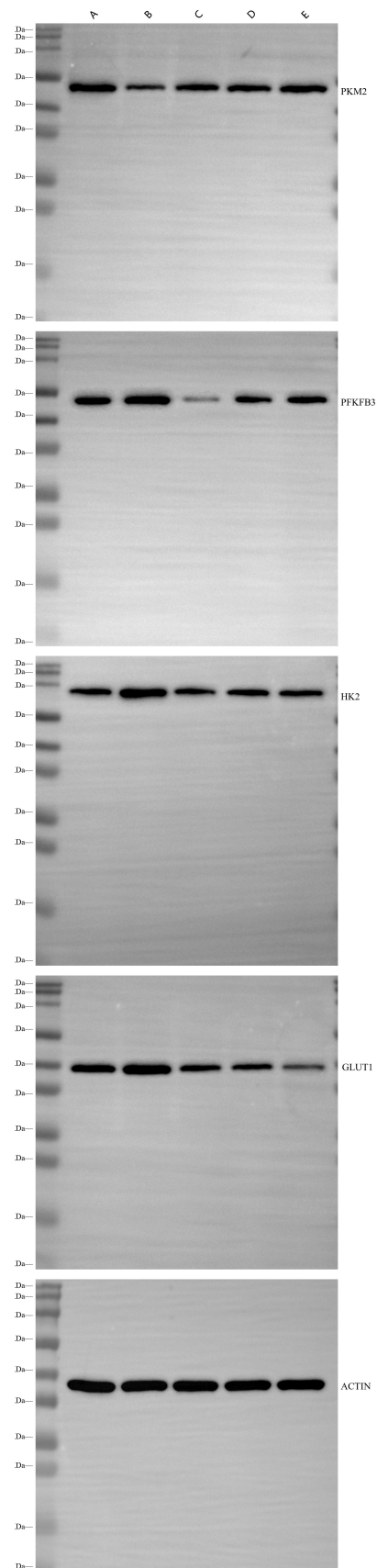

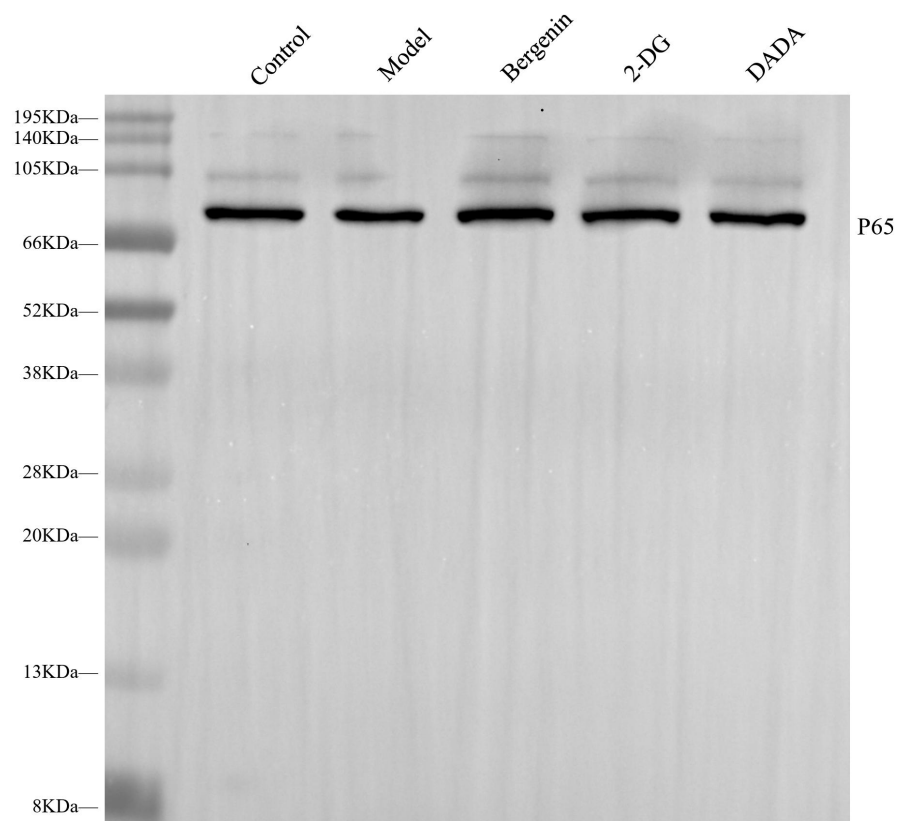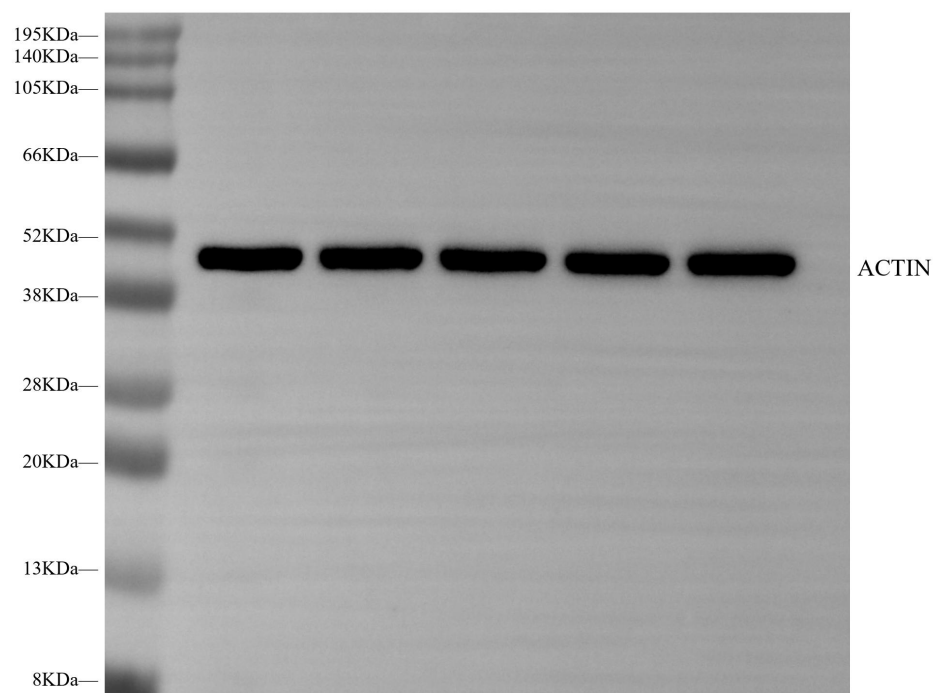

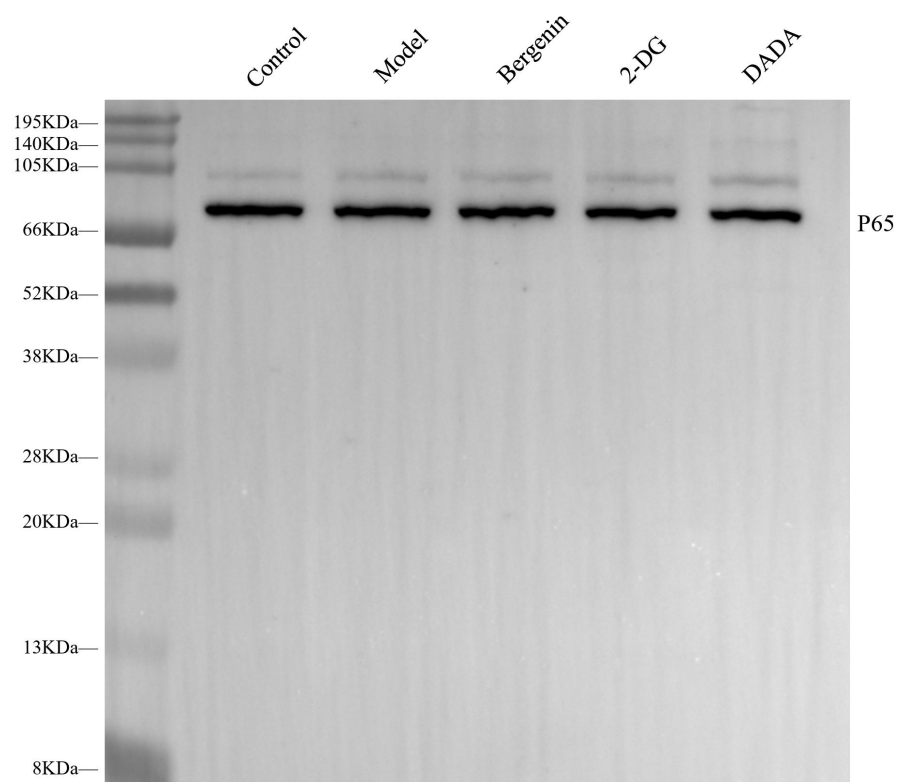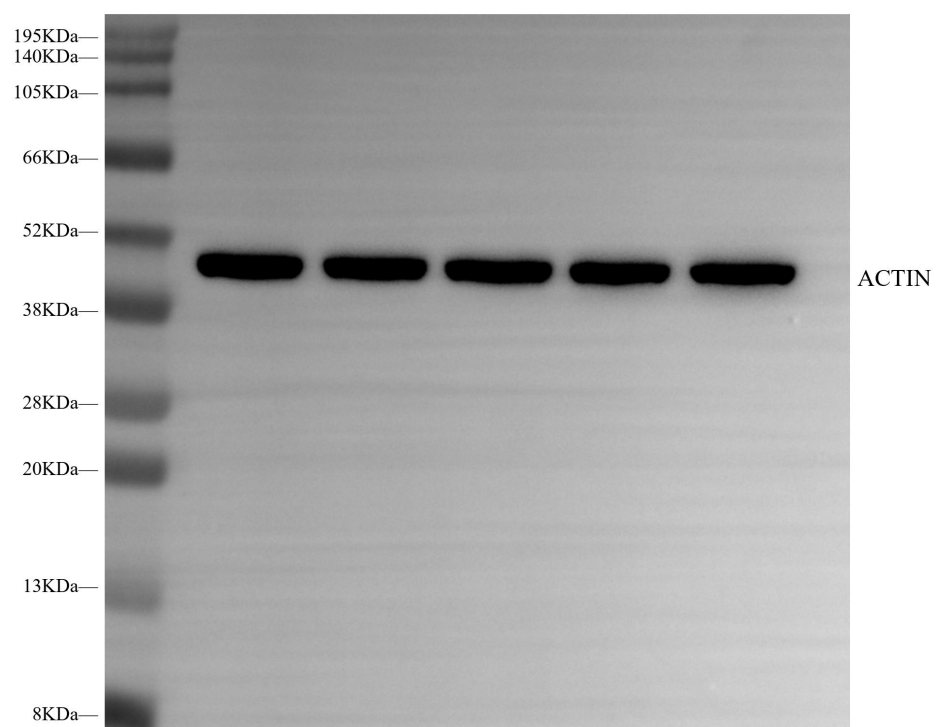

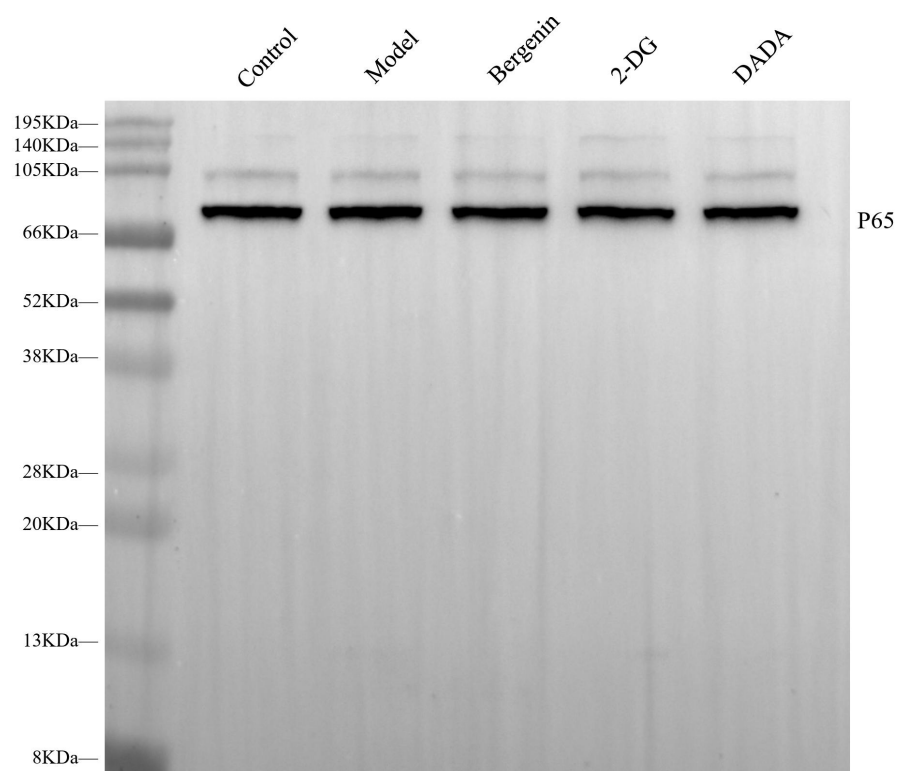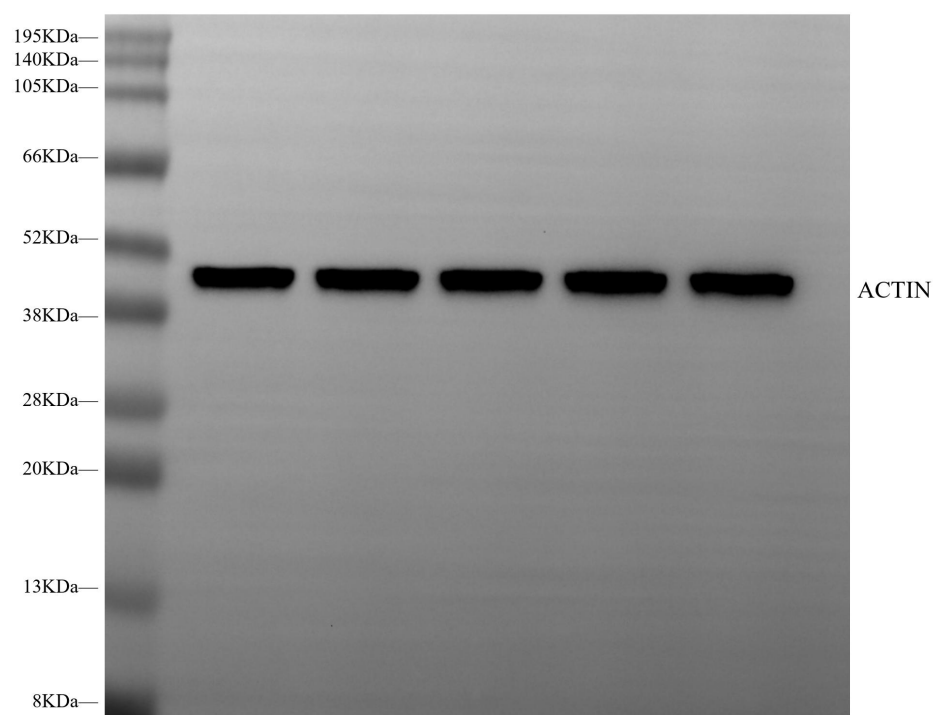

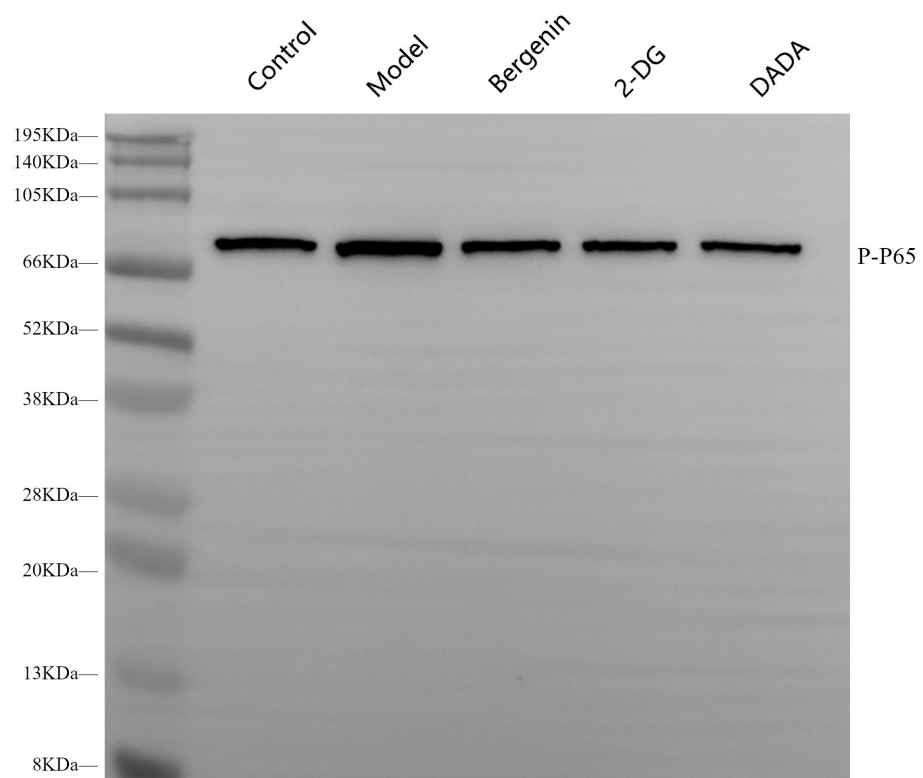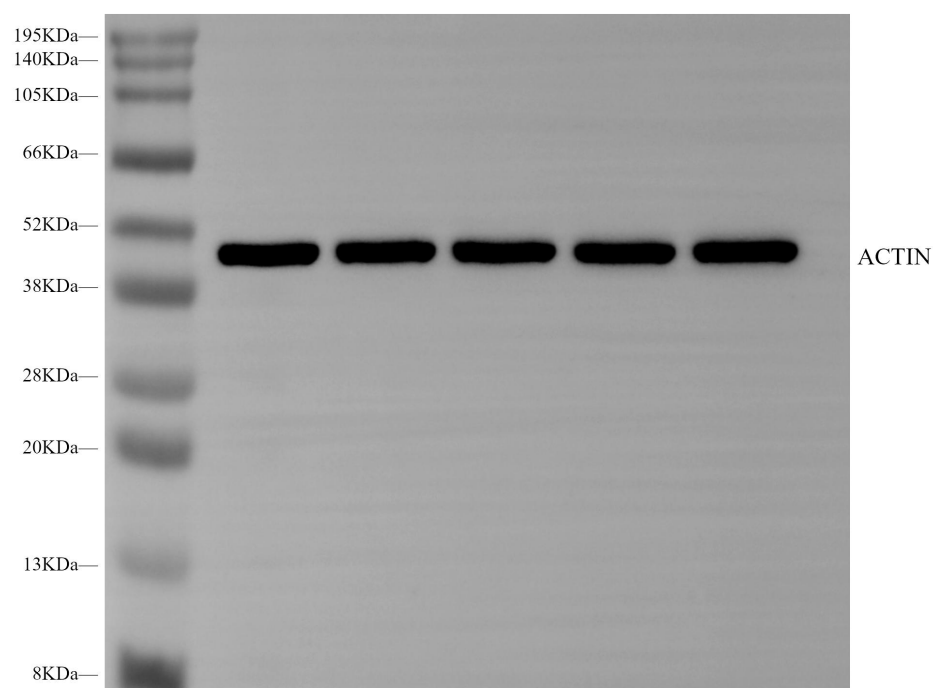

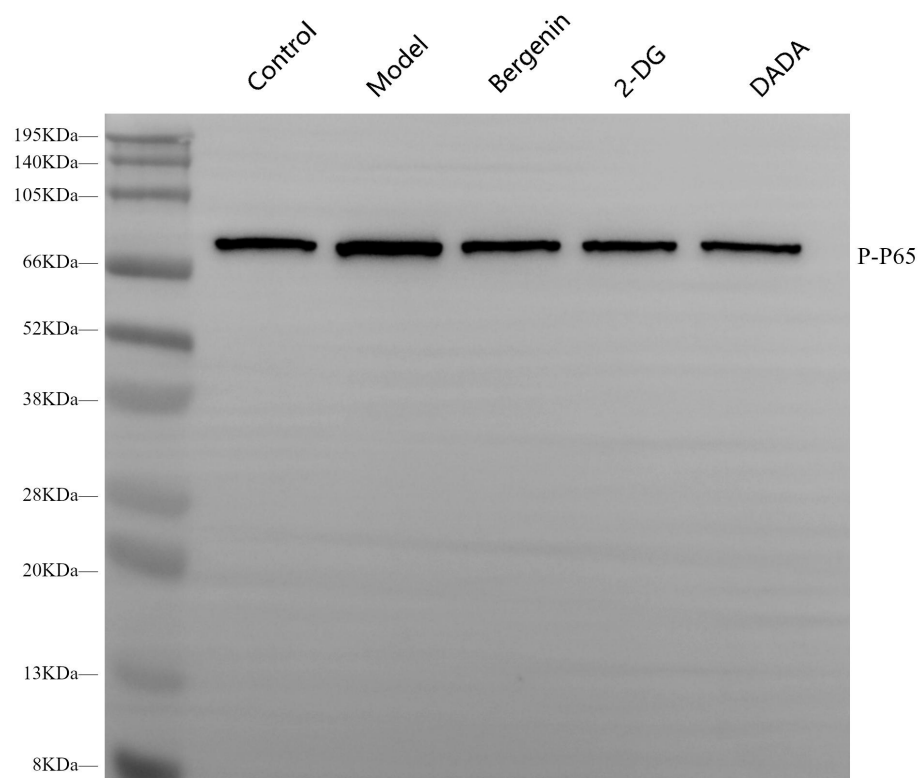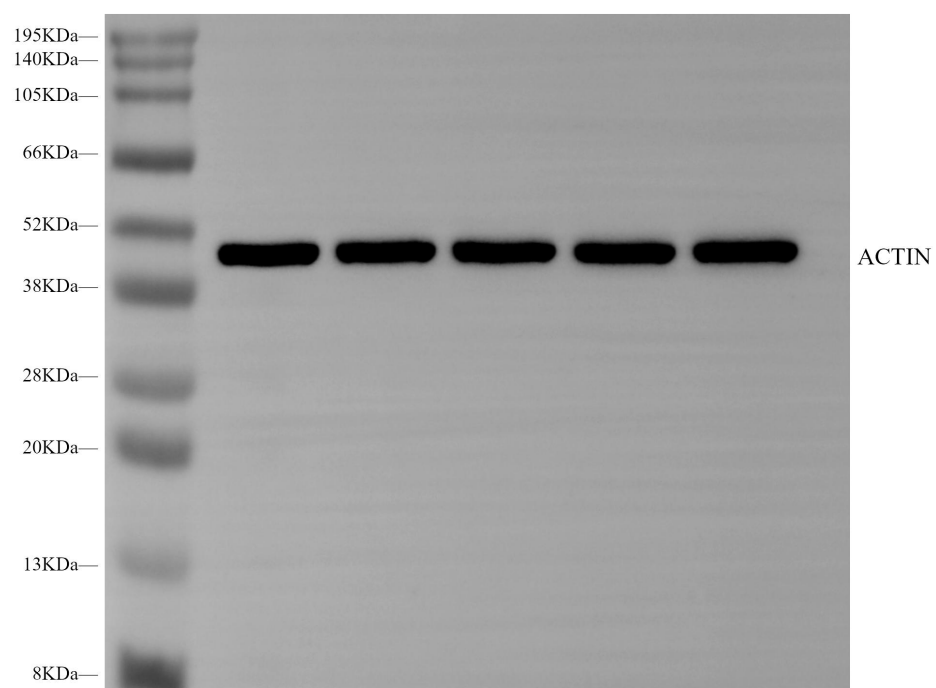

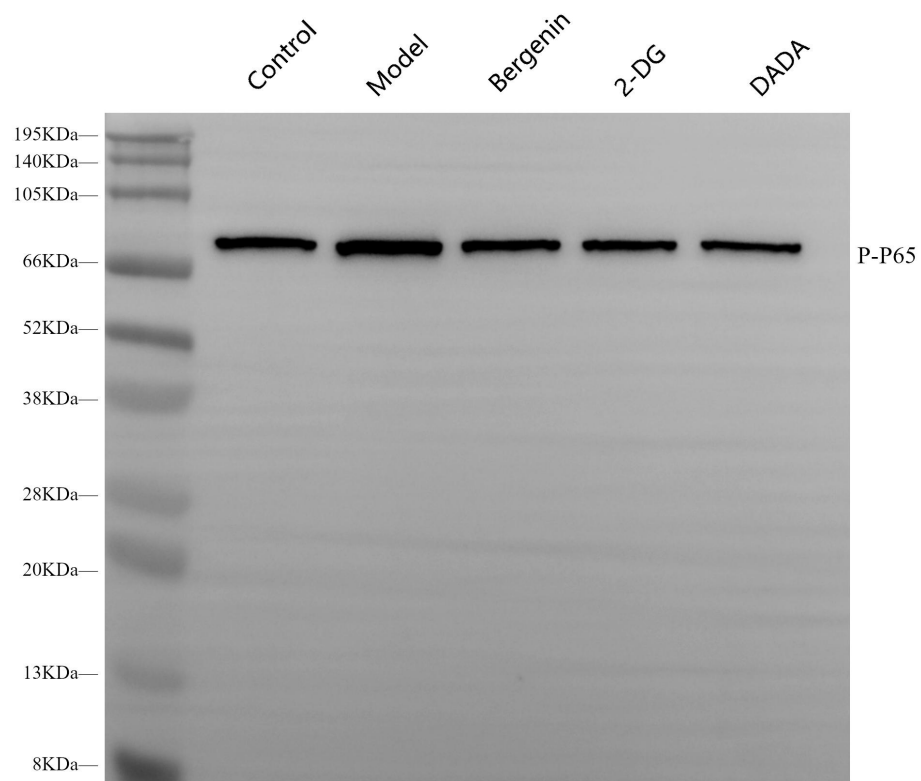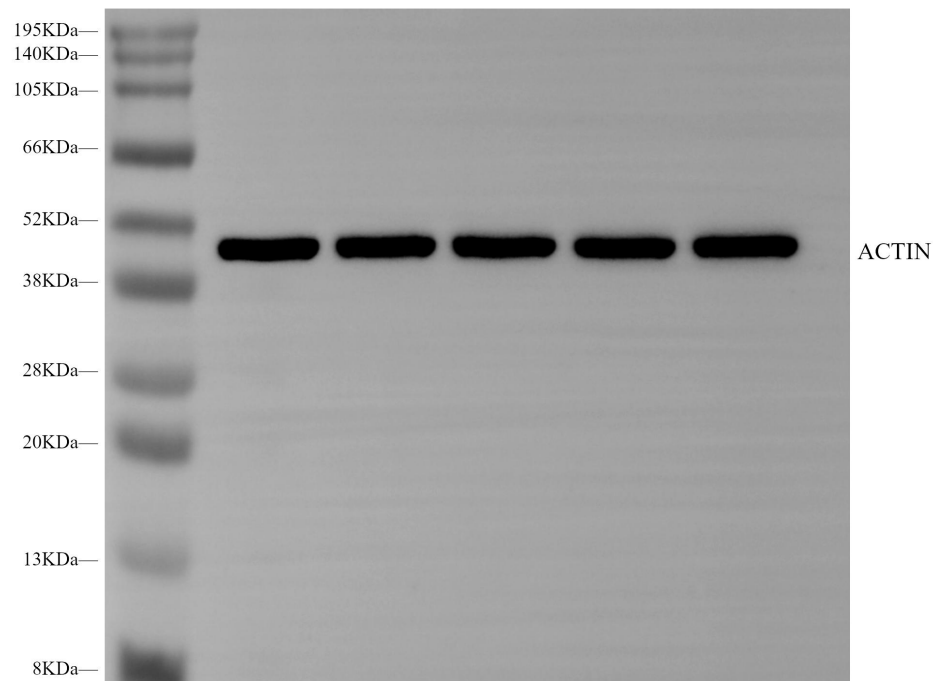

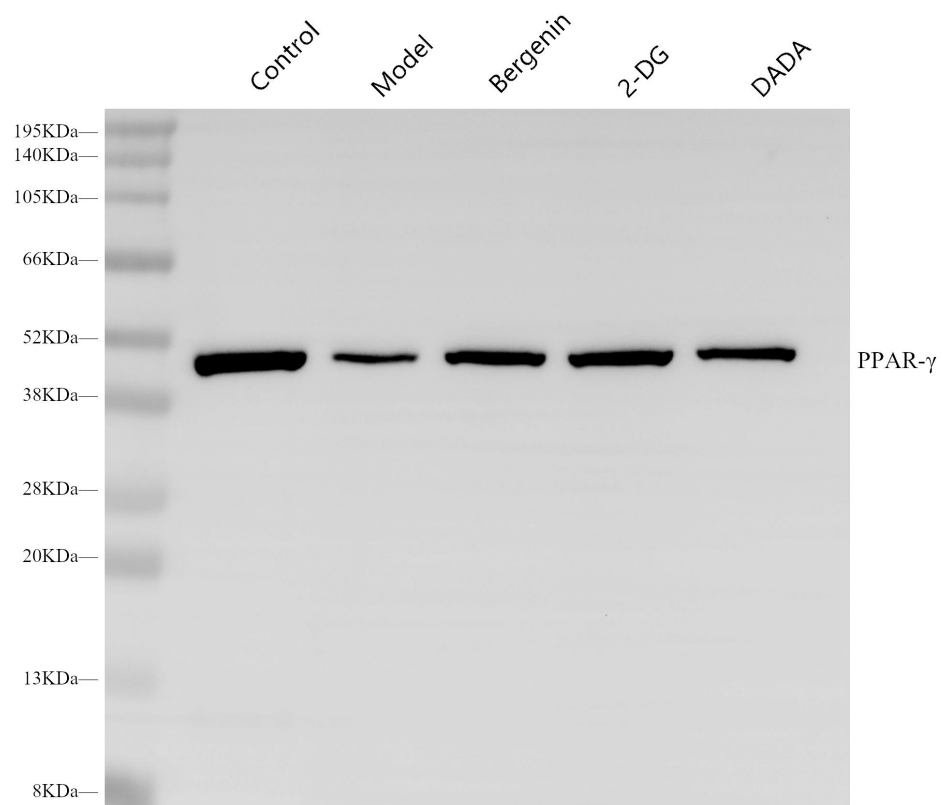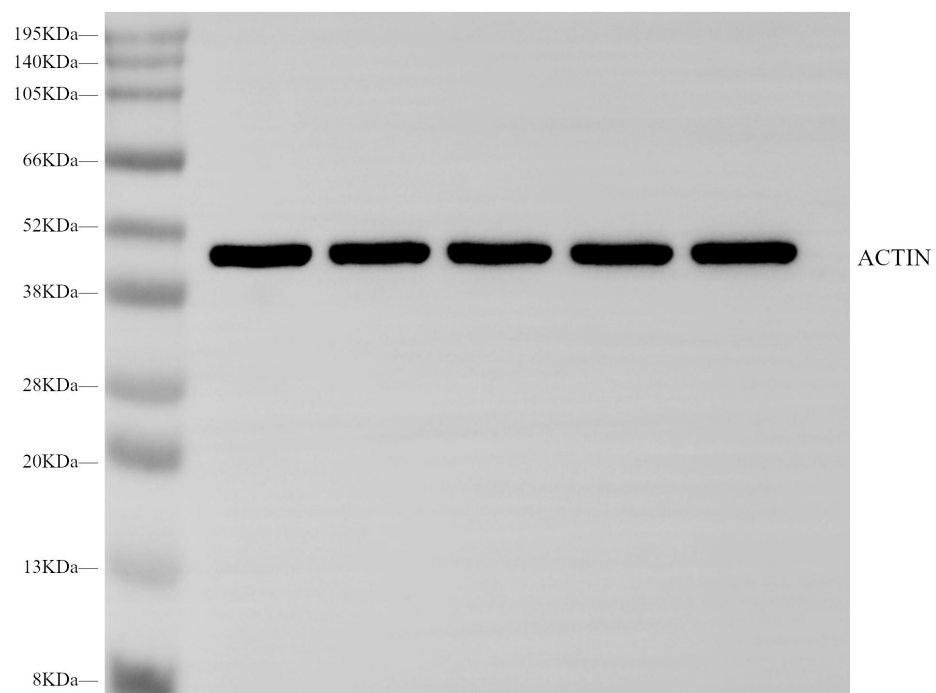

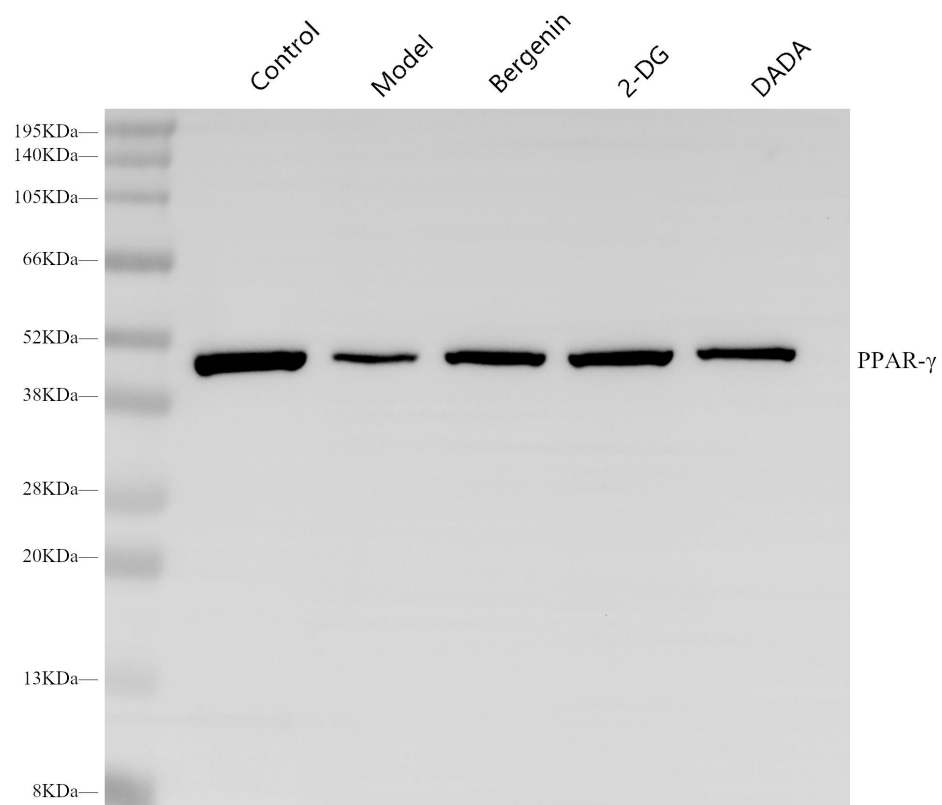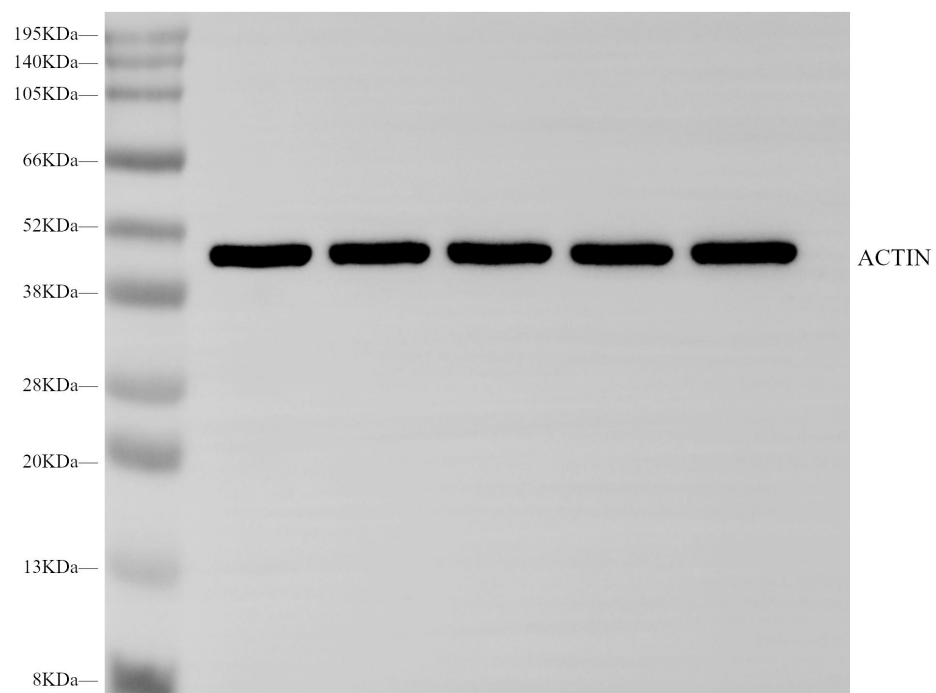

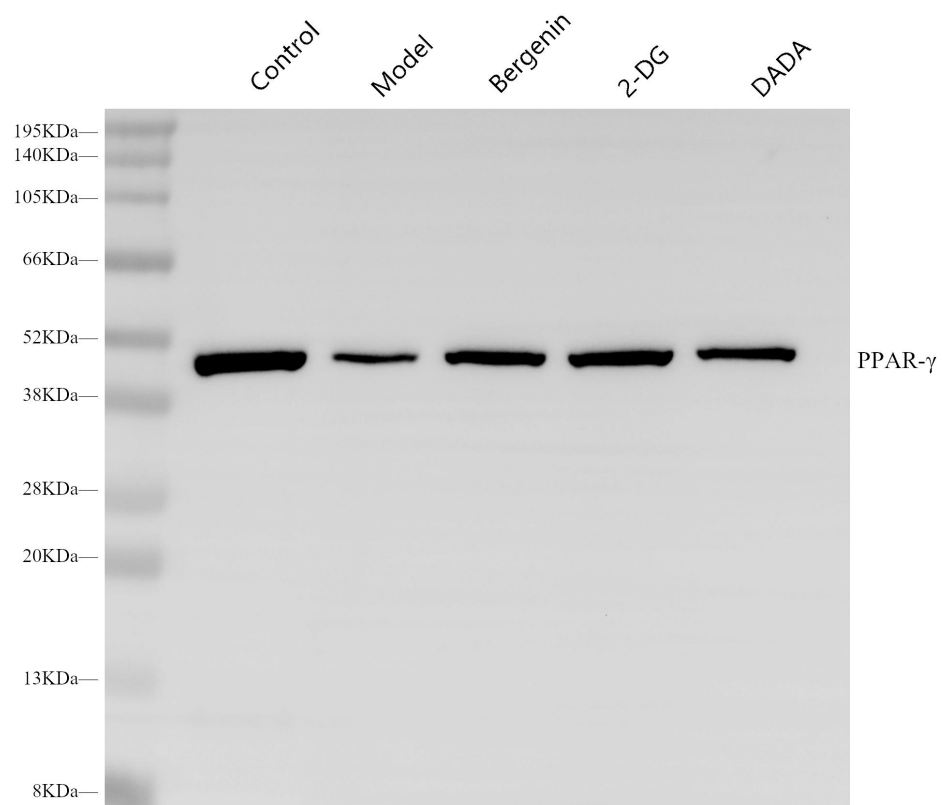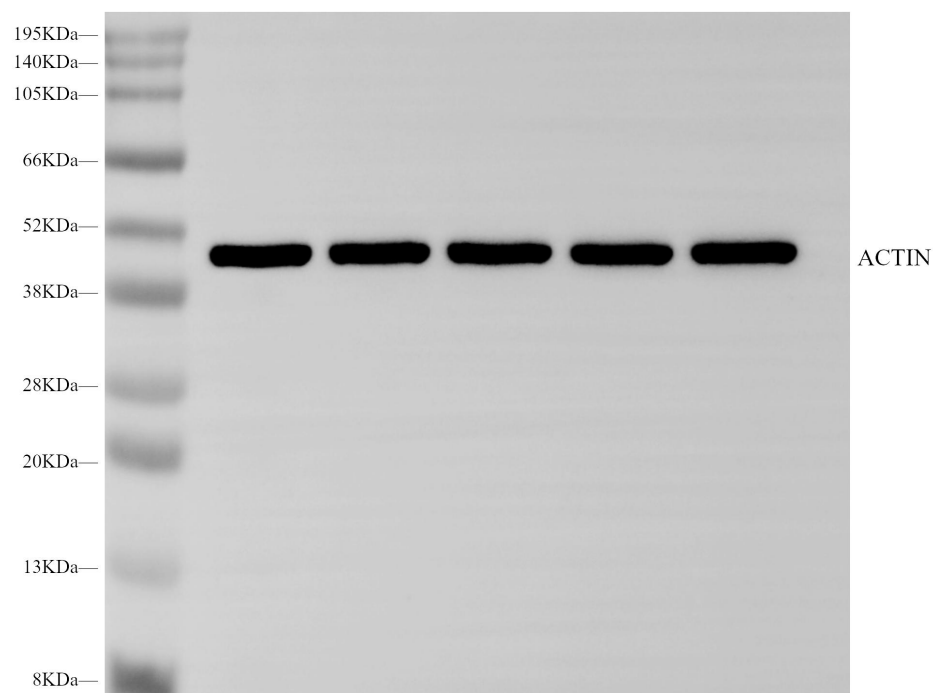

Supplement: Supplementary file 2 [file DataSheet1.PDF]
